# Discrete choice experiments or best-worst scaling? A qualitative study to determine the suitability of preference elicitation tasks in research with children and young people

**DOI:** 10.1186/s41687-021-00302-4

**Published:** 2021-03-10

**Authors:** Helen J. Rogers, Zoe Marshman, Helen Rodd, Donna Rowen

**Affiliations:** 1 Unit of Oral Health, Dentistry and Society, School of Clinical Dentistry, University of Sheffield, Sheffield, UK; 2 Health Economics and Decision Science, School of Health and Related Research, University of Sheffield, Sheffield, UK

**Keywords:** Adolescent, Preference-elicitation, Discrete choice experiment, Best-worst scaling

## Abstract

**Background:**

Ordinal tasks are increasingly used to explore preferences for health states. This study aimed to determine the suitability of two ordinal preference elicitation tasks (discrete choice experiments (DCE) and best-worst scaling (BWS)) for use with children and young people to generate health state utility values. The study explored children’s understanding, the relationship between their age and level of understanding, and how many tasks they felt they could complete.

**Methods:**

Children aged 11–16 years were recruited from a secondary school in South Yorkshire, UK. Participants were asked to ‘think aloud’ as they completed a computer-based survey that contained both DCE and BWS tasks relating to dental caries (tooth decay) health states. Health states involved descriptions of the impact of tooth decay on children’s daily lives. One-to-one semi-structured interviews were then held with participants, with use of a topic guide. Qualitative data were transcribed verbatim and analysed thematically.

**Results:**

A total of 33 children (12 male, 21 female) participated, comprising 5–6 children from each school year group. Children expressed a preference for BWS and demonstrated a better understanding of these tasks than DCE. There was no clear relationship between children’s level of understanding and age. Children felt they could manage between 8 and 10 BWS tasks comfortably.

**Conclusion:**

This study suggests that BWS tasks are the most appropriate type of preference elicitation task to value health states for children and young people aged 11–16 years to complete.

**Supplementary Information:**

The online version contains supplementary material available at 10.1186/s41687-021-00302-4.

## Background

The involvement of children and young people in dental research is now considered a priority, with calls to view them as active participants [1, 2]. It is widely acknowledged that children and young people can self-report their own health using patient report outcome measures (PROMs). However, for a PROM to be used directly in economic evaluation to inform resource allocation decisions it must be preference-based to enable it to be used to generate quality adjusted life years (QALYs). This means that there must be a value set for the preference-based measure that reflects preferences around how good or bad the health state is on a 1–0 full health-dead scale. For a child and adolescent-specific measure, the value set can be generated using preferences elicited by adults or from children and young people themselves. Since children and young people experience the health states described by the measure, it can be argued that it is their health state preferences that should be incorporated [3]. There is increasing interest and research into the comparability of health state preferences elicited from adults with those from children and young people in larger valuation surveys [3–7]. However there is little qualitative evidence on whether children and young people understand these preference elicitation tasks and find them appropriate.

Cardinal methods such as time trade-off (TTO) and standard gamble (SG) are the most commonly used techniques to elicit health state preferences, and require participants to consider trading a year of their life or the risk of death respectively. Whilst one measure, the Assessment of Quality of Life-6D (AQoL-6D), has gained preference weights using the TTO method with adolescents aged 16–17 years, in general these methods are considered to be too cognitively demanding for adolescents [8]. Furthermore, ethical concerns have been raised about using techniques that involve consideration of death with adolescents. More recently, the use of ordinal techniques, such as profile case best-worst scaling (henceforth referred to as BWS) and discrete choice experiments (DCE), have shown promise as more appropriate methods to elicit adolescent preferences [6, 7, 9–11]. Nonetheless, without the inclusion of a duration attribute due to the aforementioned ethical issues, it is difficult to anchor the values obtained using ordinal techniques onto the 1–0 full health to death scale required to determine QALYs, and the preferences of adults may need to be incorporated for this purpose.

Whilst both DCE and BWS have been successfully used in large scale valuation surveys with adolescents, it is important to acknowledge that there is little qualitative evidence available to inform the design of these surveys, for example, which age range of adolescents are able to understand these tasks, and how many tasks they can complete [5–7, 9, 10]. The feasibility of using BWS tasks with adolescents was determined in a study by Ratcliffe and colleagues, through a comparison with cardinal tasks [9]. The findings suggested that BWS tasks were more readily understood and interpretable by this population. Stevens undertook cognitive debriefing with 31 New Zealand schoolchildren aged 7–17 years as they completed tasks based upon the descriptive system from the Child Health Utility-9D (CHU9D) to determine the reliability of ordinal methods [11]. The results indicated that those aged 14 and above were able to understand pairwise DCE, but that BWS could potentially be completed by children as young as 10 years. Whilst these studies have confirmed that ordinal tasks have potential for use in preference elicitation with adolescents, it remains unclear as to which type of ordinal task is most appropriate for children and young people to complete.

The present study investigated whether DCE or BWS tasks are most appropriate for use with adolescents to value health states, within the context of the development of a child-centred condition-specific-preference-based measure for children and adolescents with dental caries (tooth decay), using computer-based DCE and BWS tasks. Preference-based measures are used to generate quality adjusted life years (QALYs), enabling the same common metric to be used to assess benefits across different interventions both within oral health and across other fields of healthcare. However, previous research has found that generic paediatric preference-based measures that are typically used to generate QALYs do not perform well psychometrically in oral health, [12] suggesting the need for a condition-specific preference-based measure. This is an important step towards addressing the limited use of QALYs in current oral health research [13, 14].

### Aim and objectives

This study aimed to identify the suitability of two ordinal preference-elicitation tasks (DCE and BWS) for use in a computer-based self-administered survey with children and young people aged 11- to 16-years from a secondary school in South Yorkshire, UK. The specific objectives of this study were to:

- Determine adolescents’ level of understanding for each type of task and consider how this relates to their age
- Identify the number of tasks adolescents are able to complete
- Ascertain which type of task (DCE or BWS) adolescents prefer and the reasons behind their preferences

## Methods

In order to address this aim, a computer-based survey was designed for secondary school pupils to complete, containing both DCE and BWS tasks comprised of health states surrounding the impacts of dental caries. Children and young people were asked to ‘think aloud’ whilst they completed the survey, which was immediately followed by a one-to-one semi-structured qualitative interview.

### Health states

The health states for the DCE and BWS tasks within this survey were based upon the classification system for a caries-specific preference-based measure. The classification system (Table 1) contains five items (*‘hurt’, ‘annoy’, ‘hard to eat’, ‘cry’, ‘kept awake’*) and three levels (*‘not at all’*, *‘a bit’* and *‘a lot’*) which were derived from the Caries Impacts and Experiences Questionnaire for Children (CARIES-QC); a child-centred measure of oral health-related quality of life specific to caries [15]. A combination of approaches was used to identify the classification system, including Rasch analysis, classical psychometric testing, involvement of children, young people and parents, as well as the team who developed the original measure. Details of this process and the child-centred validation of the classification system are described elsewhere [16].

**Table 1:** The classification system for a caries-specific preference based measure and related questions from CARIES-QC

Questions from CARIES-QC	Response levels
How much do your teeth hurt you?	My teeth do not hurt me at all
	My teeth hurt me a bit
	My teeth hurt me a lot
How much do your teeth annoy you?	My teeth do not annoy me at all
	My teeth annoy me a bit
	My teeth annoy me a lot
Do your teeth make it hard to eat some foods?	My teeth do not make it hard at all for me to eat some foods
	My teeth make it a bit hard to eat some foods
	My teeth make it really hard to eat some foods
How much do you get kept awake by your teeth?	My teeth do not keep me awake at all
	My teeth keep me awake a bit
	My teeth keep me awake a lot
How much have you cried because of your teeth?	My teeth do not make me cry at all
	My teeth make me cry a bit
	My teeth make me cry a lot

### Survey design

A survey (Supplement 1) was designed with SurveyEngine (SurveyEngine GmbH, Berlin, Germany) for young people to complete independently using a computer. The survey contained basic demographic questions, followed by questions about general and dental health.

The five questions from the classification system derived from the CARIES-QC (Table 1) were included as a warm-up exercise to familiarise participants with the health state descriptions used in the survey.

The survey asked participants to think about their teeth *today* when answering these questions. Basic information about tooth decay was provided, accompanied by a photograph of a decayed tooth. This was followed by five BWS tasks and five DCE tasks; the order of which was presented to the participant first was randomised to minimise ordering effects. The authors previously confirmed the independence of the items within the classification system for CARIES-QC-U, and the plausibility of the tasks was confirmed with active involvement of children and young people.

**Fig. 1:**
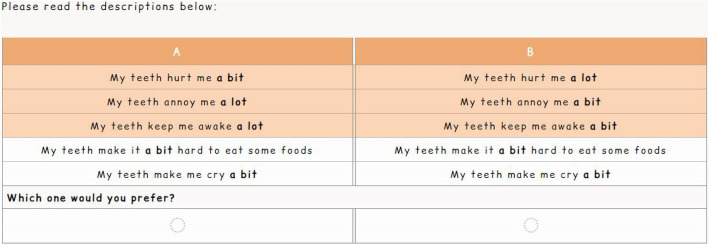
Example of a discrete choice experiment task from the survey

**Fig. 2:**
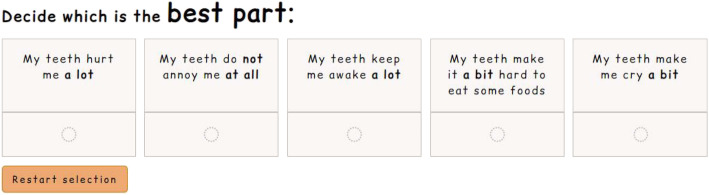
Example of a best-worst scaling task from the survey

Figure 1 shows an example of a DCE task from the survey. The pairwise design provides the participant with two alternative hypothetical health state profiles from which they select their preferred option. Figure 2 provides an example of a BWS task from the survey. The profile case design provides the participant with just one health state profile, from which they select the best and the worst feature. As this was not a valuation survey, an experimental design was not used when developing the tasks. Instead, the tasks were chosen to ensure a range of attribute/level combinations and varying complexities. All participants answered the same questions, though the ordering was randomised.

Each type of task was preceded by a ‘walk-through’ demonstrating how to answer, and a practice question. At the end of the survey, participants were asked how easy or difficult they found each task to answer and to understand, and which type they preferred. The font and colour scheme for the survey (seen in Figs. 1 and 2) were chosen in accordance with national guidance to aid participants who had specific learning difficulties and visual impairments [17].

### The interview

Interviews were conducted by one researcher (HJR), a female paediatric dentist with experience and formal training in qualitative techniques, in a private room. Participants were unaware that the researcher had a dental background but understood the research was being undertaken as part of a research degree. Participants worked their own way through the survey, but were advised that they could ask for help if they were struggling. Participants were asked to ‘think aloud’ intermittently throughout the survey, and encouraged to explain their decision-making processes when completing the tasks to allow the interviewer to determine the participants’ level of understanding. A short semi-structured interview based upon a topic guide was then conducted, following completion of the survey. The topic guide (Supplement 2) was developed iteratively, with involvement of child and parent study representatives, to explore topics such as the reasons behind participants’ preference for one type of task, and the number of tasks they felt they could manage to complete in a survey. Interviews were recorded using a digital voice recorder, and transcribed verbatim. No non-participants were present, and field notes were taken throughout.

A pragmatic methodology was considered to be the most appropriate stance for this study, as it enabled the research question to be addressed without requiring conformity to specific traditions aligned with other epistemological viewpoints [18].

### Recruitment

A secondary school in South Yorkshire, UK, was invited to participate in the study, primarily based upon the profile of the pupils with the school having above average proportion of pupils eligible for free school meals and ethnic diversity.

Study information and ‘opt-in’ consent forms were sent to the parents/carers of children from one class (comprising between 20 and 30 children) within each Year Group from Year 7 to Year 11, encompassing pupils aged 11 to 16 years. All participant-facing materials, including the survey, were developed with involvement of children, young people and parents, as members of the steering group for the overall study. As with previous studies using this ‘opt-in’ approach, a low return of signed parental consent forms was anticipated. All students whom had returned completed parental consent forms were invited to participate, providing they were able to understand spoken and written English, and hence could undertake the research with support if required. Based upon previous research, approximately 30 to 35 participants were required to reach data saturation, whereby no new codes or themes emerge, with at least five pupils from each year group to enable adequate representation [11]. No repeat interviews were undertaken.

Children were provided with an age-appropriate participant information sheet and invited to assent to take part. Interviews were conducted at a time chosen to minimise disruption to school lessons and examinations.

### Analysis

Simple descriptive statistics were conducted on all quantitative data using SPSS® software (IBM Corporation, United States). Qualitative data were organised using NVivo 12 software (©QSR International Pty Ltd) and analysed thematically by two researchers (HJR and ZM) independently, with agreement on the themes derived from the data through discussion.

Ethical approval for this study was obtained from Yorkshire and the Humber Research Ethics Committee (Reference: 18/YH/0148).

## Results

A total of 33 children (12 male, 21 female) took part in the survey and interviews were conducted between March and June 2019. All children who returned completed parental consent forms agreed to participate in the study. Participants’ ages ranged from 11 to 16 years, with a mean of 14 years (SD 1.55). The majority of participants reported themselves to be in ‘good’ general health (55%, *n* = 18) and to have no dental problems (82%, *n* = 27). Participant characteristics can be seen in Table 2.The time taken for participants to complete the survey and interview ranged from 10 to 20 min.

**Table 2:** Summary of participant characteristics

	Participant characteristics (***n*** = 33)
**Age (years)**	mean = 14; range = 11–16
**Gender**	
Male	12 (36.4%)
Female	21 (63.6%)
**Self-reported general health: in general, how would you rate your health today?**	
Very good	8 (24.2%)
Good	18 (54.5%)
OK	5 (15.2%)
Bad	2 (6.1%)
Very bad	0 (0%)
**Self-reported dental health: how much of a problem are your teeth for you?**	
Not at all	27 (81.8%)
A bit	4 (12.1%)
A lot	2 (6.1%)

The qualitative findings arising from the ‘think aloud’ completion of the survey and subsequent semi-structured interviews related to participants’ understanding, more specific comments relating to the two types of task, general suggestions regarding the survey and decision-making heuristics. These are described below.

### General findings relating to understanding

General findings surrounded adolescents’ ability to recall a single point in time, and their difficulties in understanding how to respond to the tasks.

Participants were prompted to ‘think aloud’ when completing the warm-up tasks, which comprised five CARIES-QC questions surrounding the impacts and experiences from their teeth *today.* Nonetheless, participants did not consider their teeth during that day only, and instead recalled their dental experiences from different time points through their lives:

*‘I’ve never really thought about my teeth that much apart from like when they were wobbly.’* Participant 26, 14 years old

*‘I’m thinking in like general because like my teeth don’t hurt anymore. They used to hurt a bit when I had braces on.*’ Participant 23, 15 years old

These past experiences influenced some participants’ responses to these questions:

*‘A few years ago I did have a hole in my tooth and I had to get it taken out and that really hurt a lot.’* Participant 33, 13 years old

Interviewer: *‘When you were answering that question, were you thinking about how your teeth were today or how they’ve been...’*

Participant: *‘Before … like the time they were pulling my tooth out. I was about like seven … I was crying because I was hurt and I’d had enough.’* Participant 11, 12 years old

Further to this, participants expressed signs of misunderstanding when completing the tasks. Whilst the instructions provided for the DCE tasks asked participants to express a preference between the two hypothetical states, instead participants had a tendency to select the option that most closely represented their own mouth and the dental impacts (or lack of) that they were experiencing. This suggested that they were unclear about how to respond to this style of task:

*“(I choose) B because my teeth are fine...”* Participant 1, 16 years old

*“(I chose that) because I’ve actually never had a problem with my teeth.”* Participant 7, 12 years old

Similar findings were noted when some participants completed the BWS tasks, whereby they based their responses on their own dentition. Nonetheless, this was far less common:

“*… So I’m thinking about like, my own teeth...”* Participant 20, 16 years old

Participants also struggled to understand or complete the tasks in other ways:

*“I think I’m going to go for B, because it has more bad things.”* Participant 15, 13 years old

### Specific comments regarding DCE or BWS

Children and young people highlighted a number of specific comments that related to the perceived differences in complexities of the two tasks and the ease with which they were felt to be understood. Further comments related to the extent that each type of task required the participant to make compromises, the number of alternative options to choose from, and the layout of the tasks.

Children had differing views regarding the DCE and BWS tasks. Children who stated a preference for the DCE tasks viewed their relative complexity in a positive light:

“*I think it gave me a bit more perspective on things …*” Participant 2, 16 years old

“*You can weigh up lots of things at once.*” Participant 15, 13 years old

“*You have to think more about which one you’d rather have.*” Participant 29, 12 years old

“*Well, personally I love reading so … everyone’s different but to be honest I actually like the A and B ones more than this multiple choice.*” Participant 20, 16 years old

Other children disagreed, and highlighted complexity as a key issue surrounding the DCE:

*“… it was a lot of information to, like, read and process at the same time.”* Participant 25, 14 years old

“*… (it) wasn’t really easy to understand...*” Participant 33, 13 years old

Conversely, children who expressed a preference for the BWS tasks valued their simplicity:

“*… (they were) a little bit easier to understand because you had like just less to think about.*” Participant 26, 14 years old

These children also acknowledge the reduced need for compromise required to complete the BWS tasks:

*“You don’t have to have like all the other bits which you might not sort of wanted like.”* Participant 17, 12 years old

However, not all children viewed this relative simplicity favourably:

“*I think it was more vague than the A or B ones.*” Participant 20, 16 years old

Furthermore, the increased number of options provided by the BWS tasks was considered a negative feature by some:

“*I find it easier to choose just between two rather than five.*” Participant 10, 11 years old

Having seen examples of BWS in both a horizontal and vertical layout (though all BWS questions that participants answered used the horizontal format), children expressed a preference for the horizontal format, as they found it easier to read and valued its originality.

*“Horizontal is better because then you can read across.”* Participant 10, 11 years old

*“(I prefer) horizontal just because it was like, not like, every other survey that I’ve taken so … it was just different.”* Participant 20, 16 years old

### Suggestions relating to the survey design

Children and young people made a number of suggestions surrounding the number of tasks they could complete, the inclusion of practice questions, the amount of information that was provided about tooth decay and how this information could be delivered.

Children proposed a large range in the number of tasks they could manage:

*‘Probably about five, so it’s probably about the right amount before I start losing concentration.’* Participant 19, 14 years old

*‘Well I could … I could do loads … and like I can probably do about 30 or 40, but I know a lot of people wouldn’t want to do more than 15 or 20.’* Participant 21, 16 years old

Generally between 8 and 10 tasks were suggested as being acceptable to themselves and other children they knew:

*‘I dunno* [don’t know]*, probably about 10.’* Participant 9, 14 years old

*‘I don’t know. I think I’d get through, like seven or eight of them and then* [start losing concentration] *…* Participant 24, 14 years old

Children thought they could complete more BWS tasks than DCE tasks due to their ease of processing and there being less to read:

Participant: *‘Probably more of those.’* [BWS]

Interviewer: *‘More? What makes you say that?’*

Participant: *‘ … because like I have a bit more choice and it’s not as difficult because you have read them in the previous ones, you know, like do I want to cry more, do I want to cry less.’* Participant 29, 12 years old

Participant: *‘I could’ve probably done more of those ones I think.’* [BWS]

Interviewer: *‘Yeah? What makes you say that?’*

Participant: *‘I suppose there was just less factors to like think about all at once.’* Participant 26, 14 years old

*‘I think it wasn’t the boredom that was the problem; it was just a lot of reading to do.’* [talking about the DCE tasks] Participant 16, 14 years old

Children offered some suggestions on how the survey could be improved. Children thought the walkthrough and practice question were useful, and that just one practice question would suffice.

Younger children thought some more information about tooth decay initially would be useful, whilst older children felt it was unnecessary.

*“… you could have added a little bit more information of why it happens and what you can do to prevent it.”* Participant 6, 11 years old

*“I think I kind of already knew that sort of stuff... because we’ve learnt about it before.”* Participant 9, 14 years old

Children thought they forgot that they were thinking about tooth decay towards the end of survey and made suggestions on how to counteract this.

*“If there’d been a sort of reminder in the middle of the quiz …”* Participant 14, 14 years old

### Decision-making heuristics

The predominant issue relating to decision-making was that participants found it difficult to view the scenarios as hypothetical, and attempted to relate the health states (particularly in the DCE tasks) to their own experiences, as discussed previously. Nonetheless, the authors gained further insights into participants’ decision-making processes, through review of the interviewer field notes and transcripts.

For some participants, their decision-making processes were limited by the extent to which they understood the task. The researchers identified that this improved as the participants became more familiar with the task. For example, following the first DCE task, Participant 8 (14 years old) stated:

“so I think I’ll probably choose this ‘cause this is, like...I don’t know”.

After the second DCE task they stated:

“I think I’ll choose B. Even though...I mean, it’s hurting a lot but...I don’t know. I’ll just go with it.”

After the third task they said:

“So...I’ll go with A. Yeah, ‘cause the teeth don’t hurt or...yeah. It’s over. I don’t know. I don’t know, like...”

Yet after the fourth task the participant began to be able to justify their decision:

“I think I’ll go with this one because it’s only a bit annoying.”

This demonstrates how decision-making heuristics may improve with practice, but also highlights how participants may simply choose any option if they do not understand the task. The perceived complexities of the DCE tasks may have contributed to this occurring more frequently than with the BWS tasks.

When completing DCE tasks, participants had a tendency to simplify the decision-making process by comparing the number of statements of different severity levels (‘a lot’, ‘a bit’ and ‘not at all’) within the two health state profiles. This occurred to a lesser degree (as demonstrated by the previous quote) when participants completed the BWS tasks. For some, this heavily influenced their response:

*“Probably this one, because it’s 'not at all' on most of them.”* Participant 14, 14 years old

This had less of an influence on others, whom incorporated their own preferences into the decision:

*“On here, there’s more ‘a lots’ than ‘bits’ on number A- on statements A, but then on statements B, the one ‘a lot’ is hurting, so I think I’m going to go for B.”* Participant 15, 13 years old

Participants described more rational decision-making processes when completing the BWS tasks:

“I’d have to think that annoying a lot … would be really hard to eat...oh, so those aren’t good. So the best would probably be not making me cry at all.” Participant 29, 12 years old

Others expanded on this, giving personal justifications for their decisions.

Participant: “… and probably keeping me awake”

Interviewer: And why do you think that was the worst bit?

Participant: “Because I love my sleep.” Participant 9, 14 years old

Some struggled to articulate the reasons for their decision, though appeared to have put some thought into it:

Participant: *“It’s the best because it’s the only ‘not at all’, and then between- to the worst, either keeping awake a lot or making me cry a lot, I think I’ll probably choose cry.”*

Interviewer: *“Why do you think that one is the worst one?”*

Participant: “*Because it’s like emotional, so it’s a bit- it’s different to being kept awake, because that’s more, yeah, that’s like the whole thing.”* Participant 18, 12 years old

There was some evidence that participants were able to trade off different attributes and/or levels for both types of task in this population:

*“I’d say the best one is ‘not annoying you’ because if you’re not annoyed then you get to focus and concentrate on everyday tasks. But if your teeth are keeping you awake a lot, then you’re not going to be able to focus because if you have lack of sleep, you might sleep in school and things like that, so that would bad for your overall health because you need to sleep as well, so yeah.”* [responding to BWS task] Participant 19, 14 years old

*“I’d probably choose B because even though your teeth are hurting more, you’re less annoyed about them and you can sleep better. So I think that even if they didn’t hurt as much, if you’re not sleeping then I feel like that would affect you more than if they’re hurt but you could sleep.”* [responding to DCE task] Participant 19, 14 years old

## Discussion

To the authors’ knowledge, this is the first study to use a qualitative approach to compare the use of DCE and BWS to elicit health state preferences from adolescents using a computer-based survey. The findings of this study suggest that children and young people aged 11–16 years old are able to understand and complete between 8 and 10 tasks as part of a valuation survey. From the perspective of children and young people, best-worst scaling tasks are the most appropriate type of ordinal preference-elicitation task for this age group to complete.

The findings from this study suggest that children and young people have a better understanding of, and ability to complete BWS tasks than DCE tasks. This relates to the relative cognitive simplicity of BWS tasks, given that there is less compromise required in completing these tasks, and a reduced quantity of text to read, comparatively. This is supported by the aforementioned pilot study which found that younger children aged 10 to 13 years were able to complete BWS tasks but struggled with DCE tasks [11]. Whilst the present study did not identify this relationship with age, it does confirm that young people find BWS to be more straightforward than DCE. Whilst BWS were initially considered to be less burdensome for an adult population also, a think aloud study has since reported contrasting results, whereby adults found the DCE tasks to be easier to complete than BWS [19, 20]. Interestingly, adults also expressed a preference for DCE tasks, compared to BWS, unlike the adolescent population in the present study [19]. There are likely to be important differences in the cognitive abilities, decision-making processes and preferences of these two groups, which reinforces the need for researchers to use the preference-elicitation task most suited to the population in question.

A number of researchers have raised concerns about the use of BWS tasks in valuation exercises to determine utilities, citing both theoretical and technical reasons, as well as a lack of research surrounding their limitations [21, 22]. Whilst acknowledging this, the authors of the present study consider the necessity of gaining high quality data from participants, through the use of a valuation task that they can better understand, to outweigh these issues. Furthermore, the use of cardinal preference elicitation techniques, such as standard gamble or time trade-off, has been deemed inappropriate for use in this population as they require the respondent to consider risking death or trading years of their life respectively. This means that if the adolescent preferences are to be used to generate value sets for preference-based measures, an alternative ordinal technique is required to elicit the preferences of children and young people. Nonetheless, a key limitation of eliciting preferences using BWS (or DCE without a duration attribute, as used in the present study) is that they cannot be anchored onto the 0–1 dead to full health scale required to generate QALYs. For this to occur, it is necessary to rescale the estimates using values obtained from an external cardinal task such as SG, TTO or DCE with a duration attribute, which may need to be sought from an adult population in light of the complexity of these tasks, and ethical concerns due to the mention of death.

Whilst a dominance test was considered for this survey, it was not possible to compare the pass rates for the two types of task, predominantly due to the difficulties in interpreting such a test for BWS tasks when used to elicit preferences for health states. This is because when an individual decides which attribute they consider to be the worst, this is informed not only by the severity of the item (*‘not at all’*, *‘a bit’*, *‘a lot’*) but also how the participant perceives the impact of the attribute to be on their quality of life. This means that whilst it is possible to determine whether respondents have correctly determined the best attribute (i.e. with the severity level of *‘not at all’*), it is not always possible to determine whether the choice of the worst attribute is irrational. Whilst there is no established precedent for the interpretation of a dominance test for the BWS task, this should not justify the dismissal of the use of BWS tasks to elicit preferences for health state valuation. Nonetheless, these difficulties in interpreting the findings from the dominance test limited the extent to which the present study was able to compare objectively how well the DCE and BWS tasks were understood.

This study may have also, indirectly, highlighted a potential issue surrounding the use of dominance tests for the DCE. The pilot study by Stevens reported that younger children had a tendency to select health states that most closely represented their own health [11]. This fits with the present study, which demonstrated that children and young people had a predilection to choose the option that was most like their own teeth. Interestingly, this finding was predominantly observed in relation to the DCE tasks and was not restricted to younger children only.

The majority of this sample reported few problems with their teeth, and hence through completing these tasks by choosing the profile that was most similar to their own health state, young people were unconsciously still choosing the best option and hence passing the dominance test. This is an area that may benefit from further research to determine whether this also occurs in other adolescent groups and potentially adult populations. A number of issues have been highlighted in recent literature surrounding the use of dominance tests, particularly the lack of a consensus on how to account for those who fail the test during the analysis [23]. Some studies use only the data from participants who pass the dominance test for analyses, though the present study would suggest that the assumption of a participants’ rationality or their understanding of the tasks based upon this alone may be inappropriate [23].

This study has also raised the possibility of a much wider issue in asking children and young people to self-report their own health. Patient-reported outcome measures of health-related quality of life are administered at multiple time points predominantly before, during and after delivery of an intervention. It is the difference in utility assigned to each health state experienced by an individual at these time points that can be used to determine the QALYs gained or lost. The present study found adolescents were unable to select their responses considering their teeth in relation to ‘today’, and instead were referring to other impacts and experiences surrounding their dentition at other time points in their lives, particularly where they had suffered dental problems. Similar observations were noted by the developers of the original CARIES-QC instrument (see Supplement 3), who decided to avoid asking participants to consider a fixed point in time when responding to questions. This potential inability to focus on the present day when self-reporting their own health could affect the quality of data gained from children and young people to generate QALYs. Whilst this may have been observed here because the sample was identified from the general population and participants were not knowingly experiencing problems with their dental health, it is possible that similar findings would be observed if the sample were reporting on their health in general. Further research is encouraged.

The think aloud nature of this study offered a number of insights to the development of the valuation survey for CARIES-QC-U, primarily leading to the use of BWS tasks for the elicitation of preferences from adolescents. The design and content of the survey, including the number of tasks for adolescents to complete, were based upon findings from the interviews and suggestions made by participants. Adaptation of the wording of the instructions to participants on how to complete the tasks was considered to reduce the likelihood of adolescents reflecting upon different time points in their lives to relate the health states under consideration to their own experiences. This research greatly benefitted from the involvement of children and young people, whom have since considered and selected alternative wording for the instructions above the tasks to be used in the valuation survey (see Supplement 4).

This study has researched an important methodological area that is particularly relevant given the increasing interest in health state valuation with children. A key strength of this study is the extent of involvement of children and young people, not only as active participants, but also as PPI (patient and public involvement) representatives and as part of the steering group. A further strength is that the interviews with children were undertaken by a researcher with formal training and experience in qualitative techniques, alongside expertise in communicating with children and young people.

A notable limitation relates to the condition-specific nature of the classification system. Whilst directly relevant for this body of work, which focusses on the development of a preference-based measure for children with dental caries, it is not known whether this methodology would produce similar findings if repeated in the context of general health, or other specific conditions. As with all qualitative research, the present study was conducted within a specific population and hence the generalisability of findings beyond this population may be limited. Lastly, the credibility of the qualitative findings could be questioned as the interpretation of the data was not relayed back to, and discussed with, the original study participants. This did not allow confirmation that the data had been interpreted as intended [24].

Future research should further investigate the value of dominance testing, and the ability of children and young people to recall their health at a specific point in time.

## Conclusion

Children and young people aged 11- to 16-years old are able to understand and complete 8–10 BWS tasks as part of a health state valuation survey. From the perspective of children and young people in this study, best-worst scaling tasks are the most appropriate type of ordinal task for this population to complete.

## Supplementary Information

**Additional file 1.**

**Additional file 2.**

**Additional file 3.**

**Additional file 4.**

## Data Availability

Anonymised data are available upon reasonable request to the authors.
