# Supplementary figures and images for: Discrete choice experiments or best-worst scaling? A qualitative study to determine the suitability of preference elicitation tasks in research with children and young people
Source: J Patient Rep Outcomes. 2021 Mar 10;5:26. doi: 10.1186/s41687-021-00302-4 (PMC7947050; doi:10.1186/s41687-021-00302-4)

####
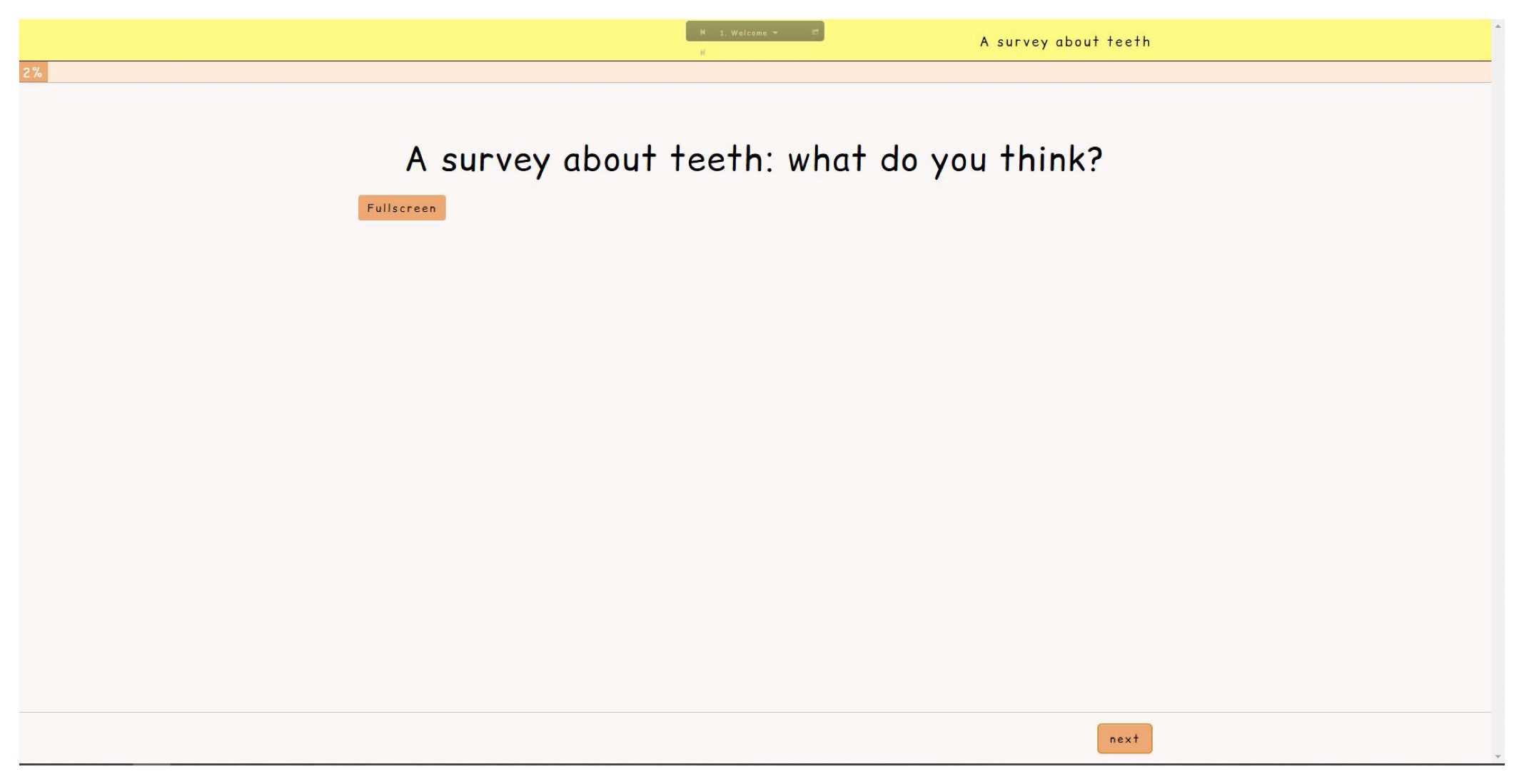


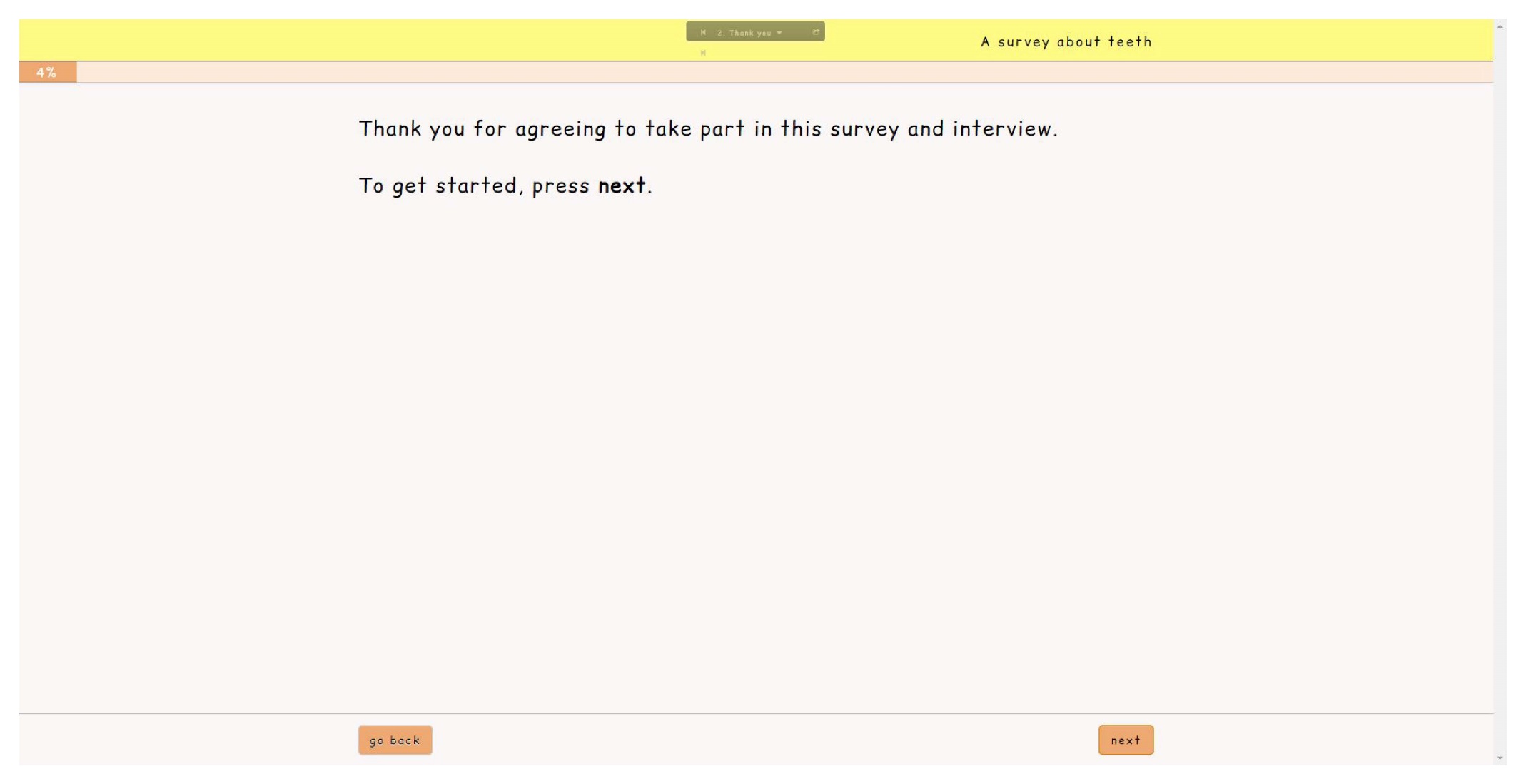

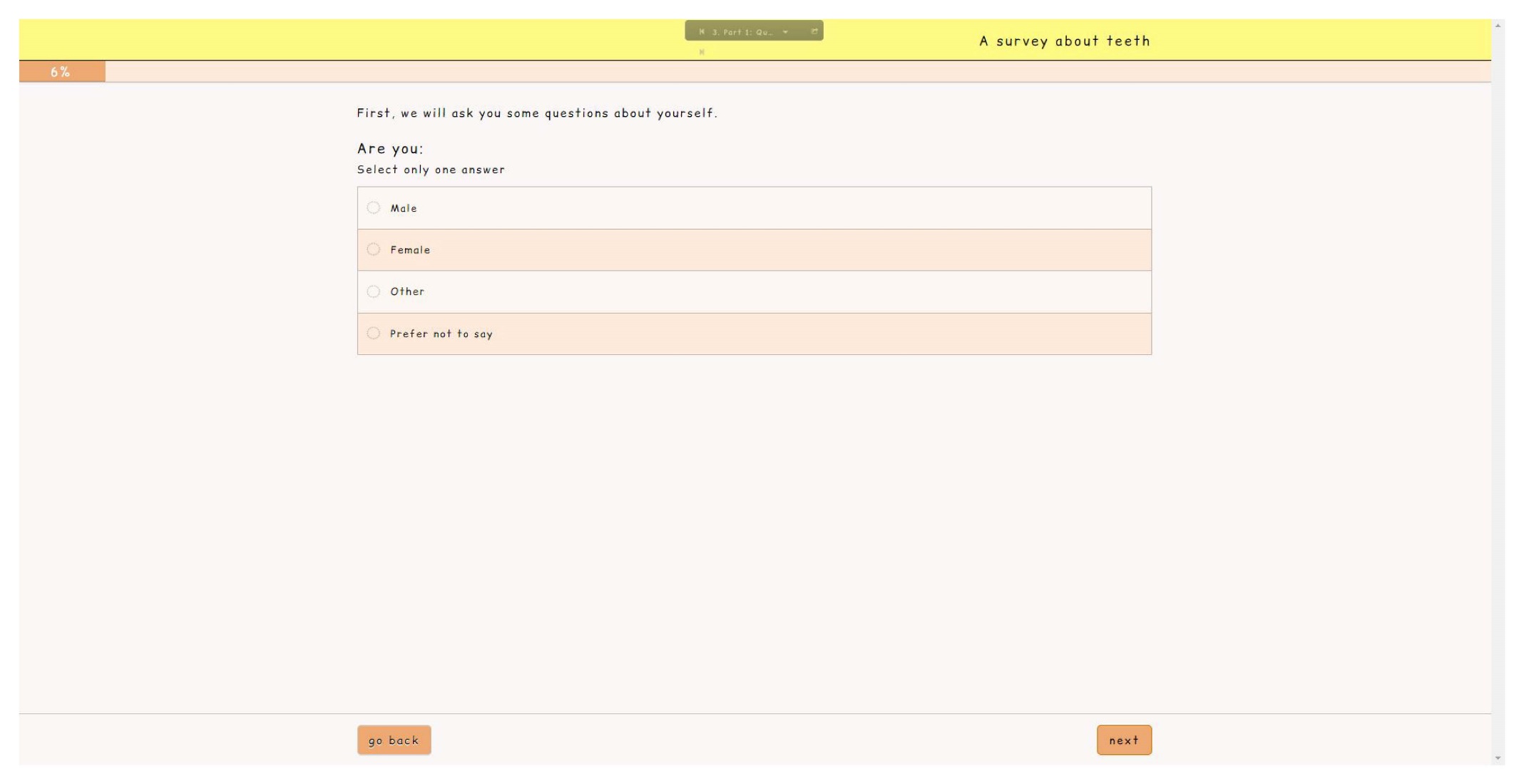

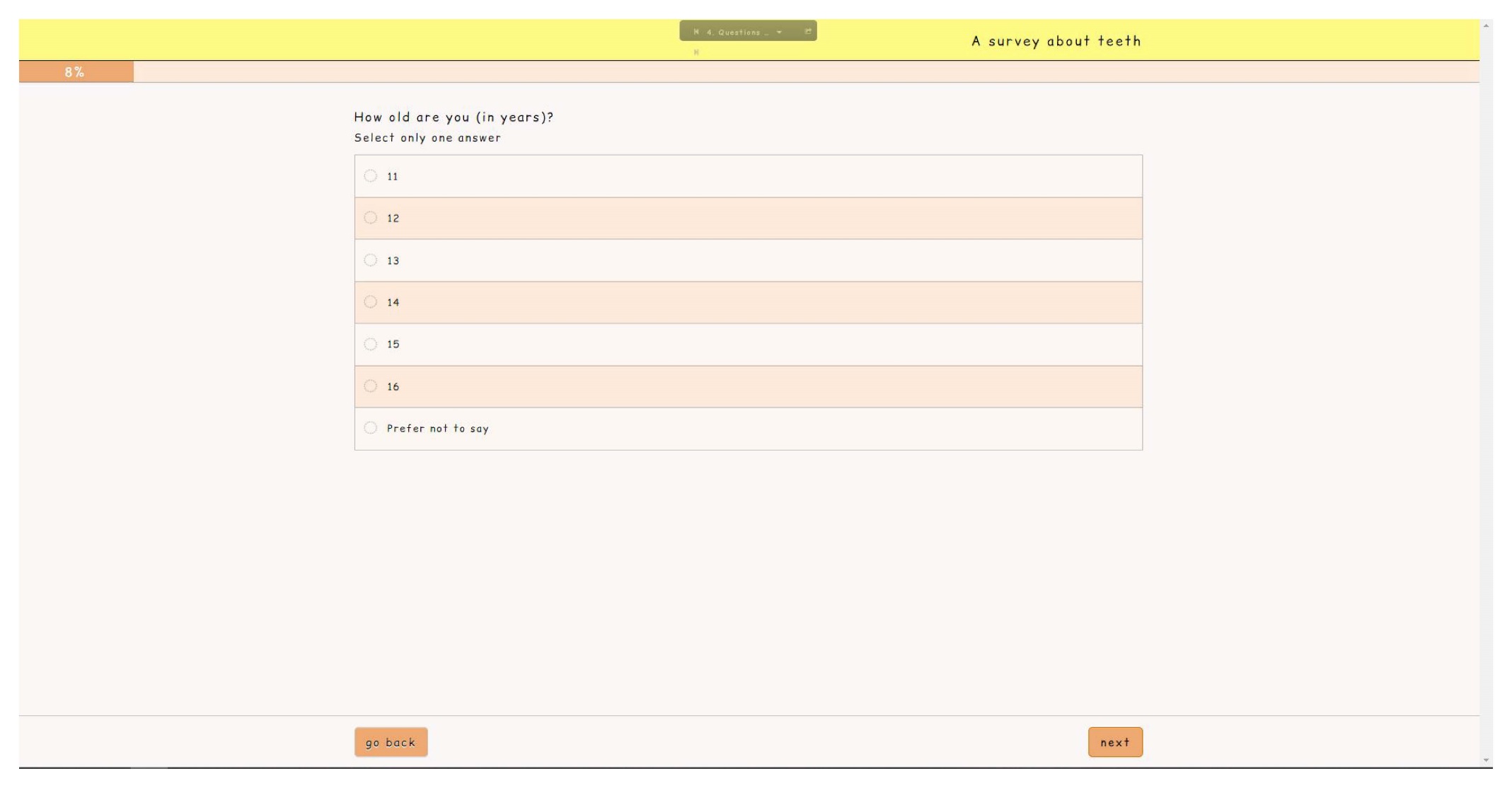

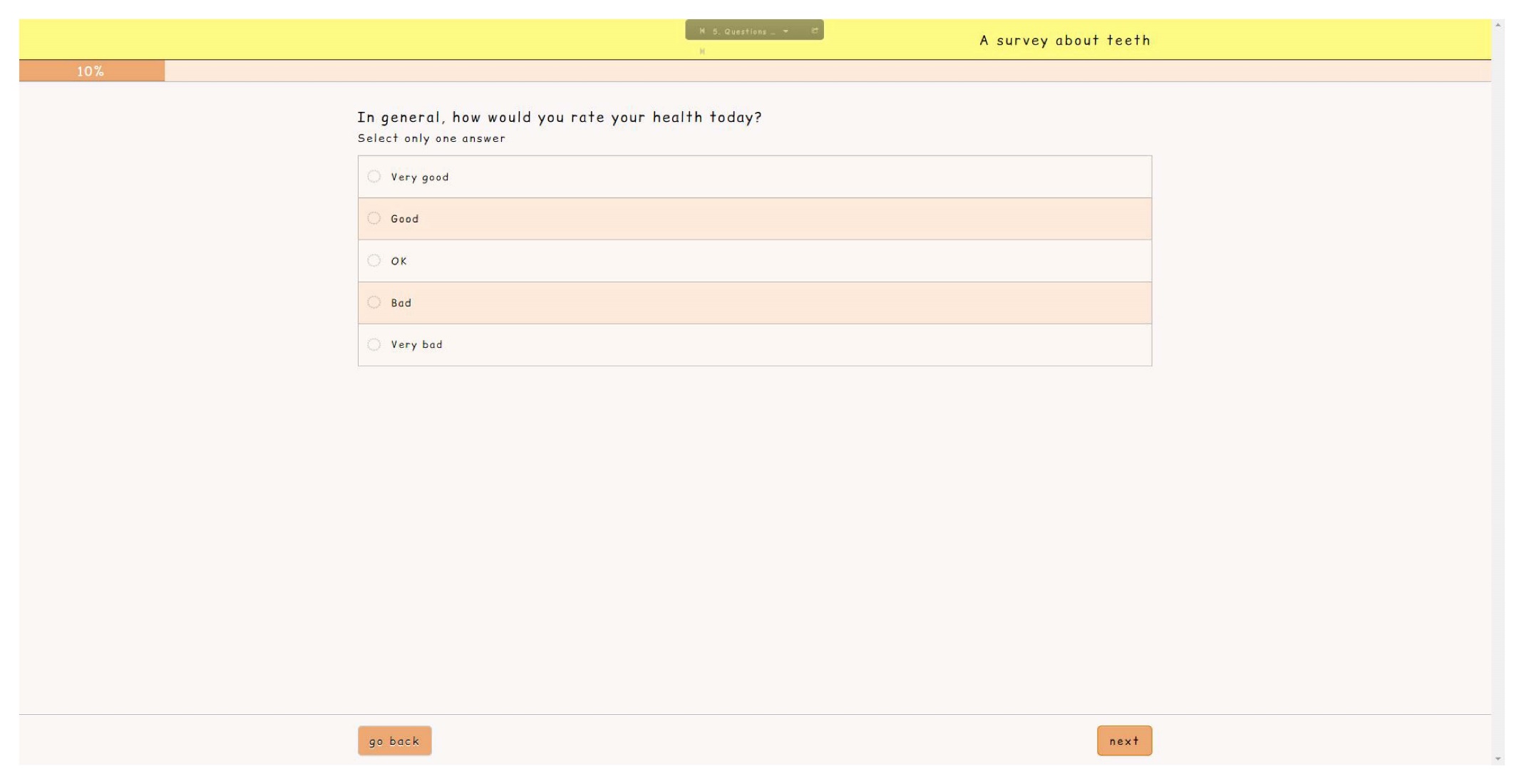

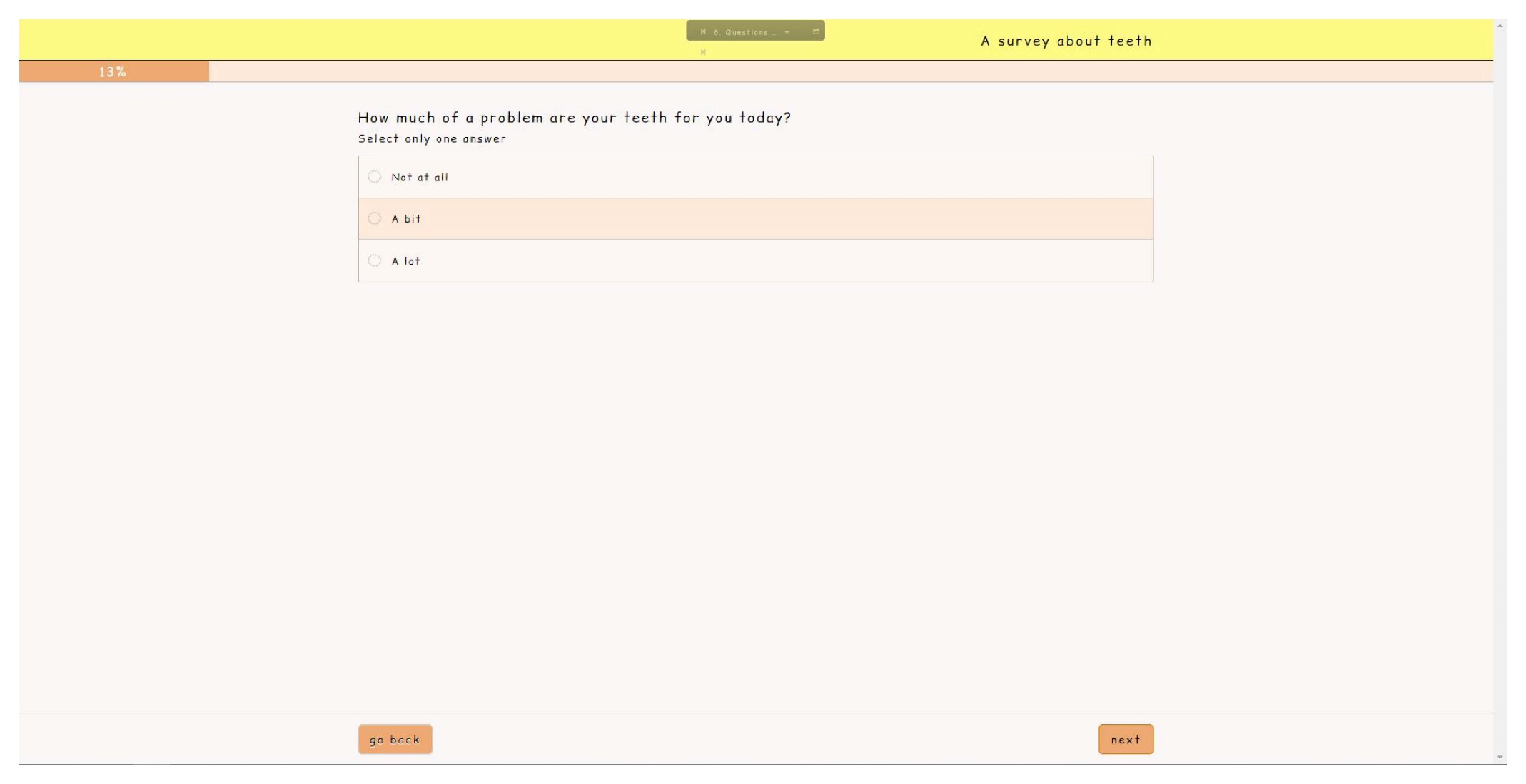

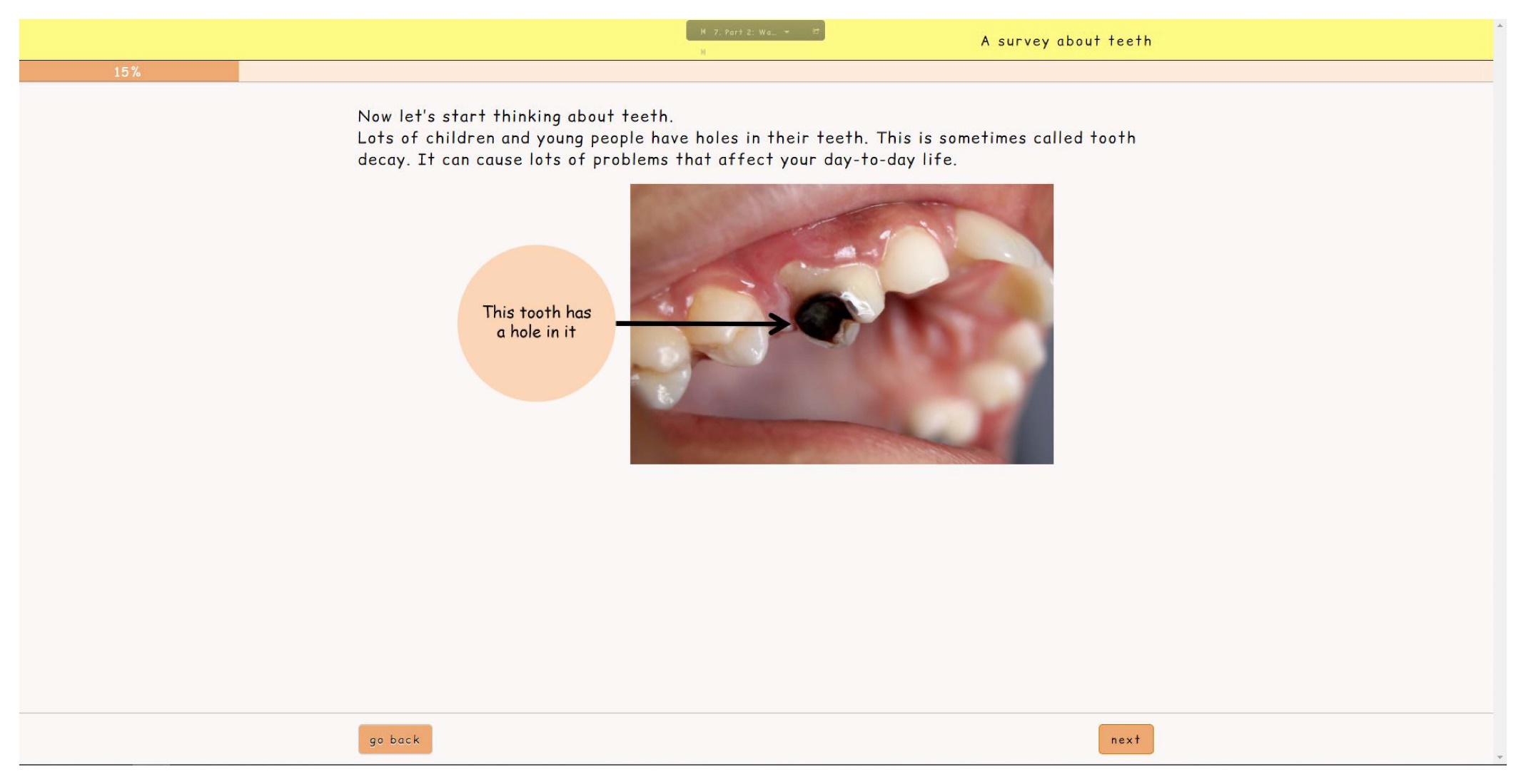

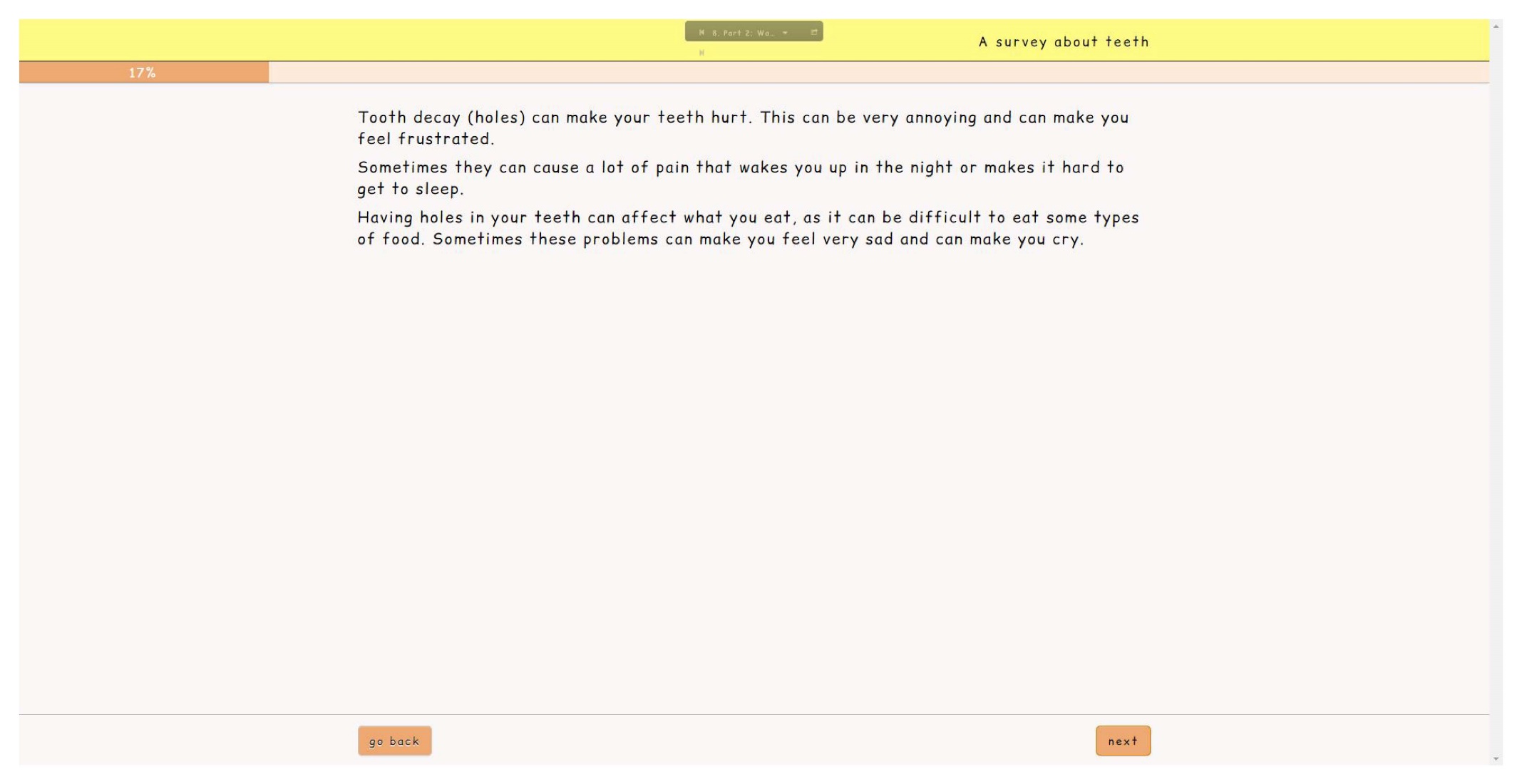

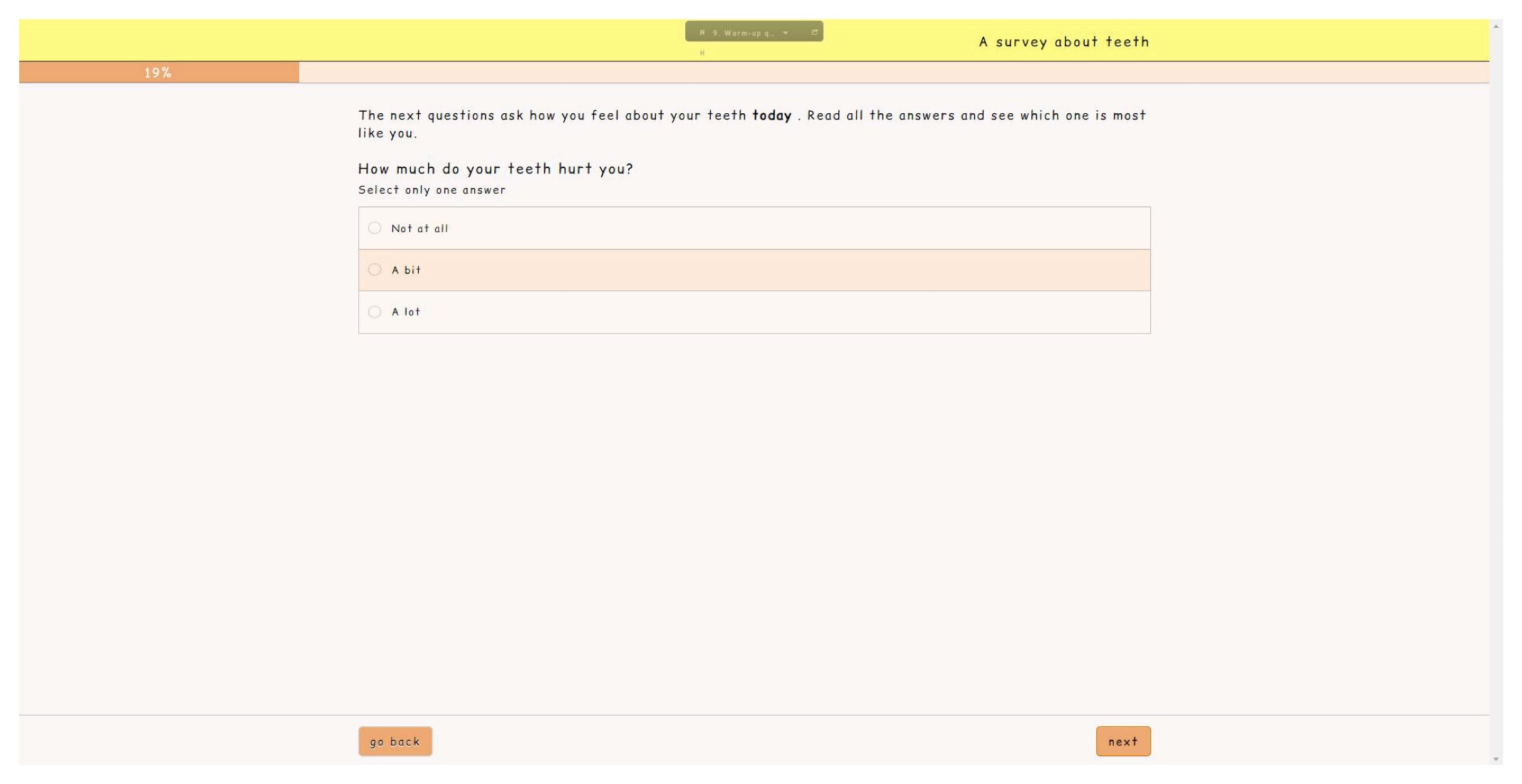

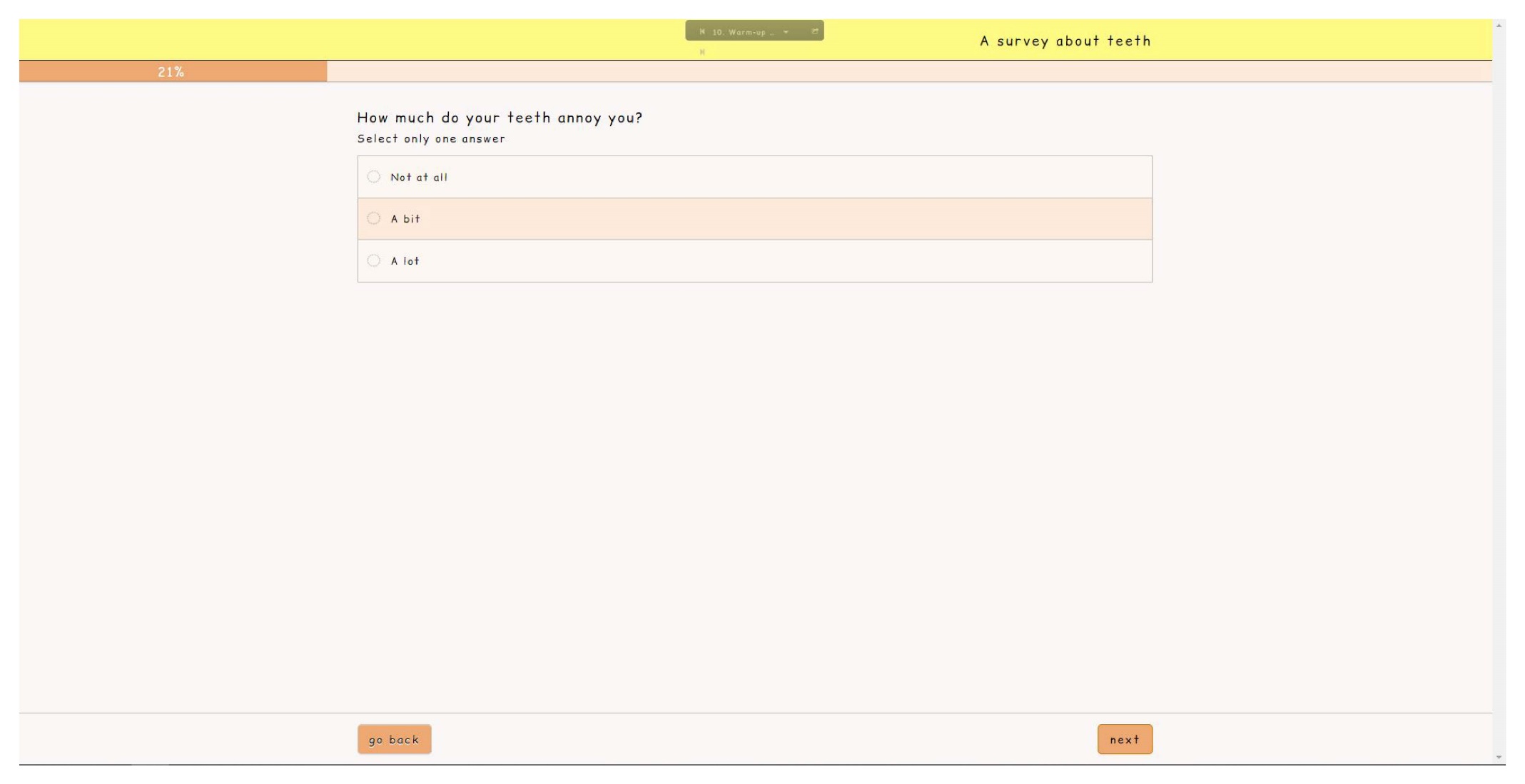

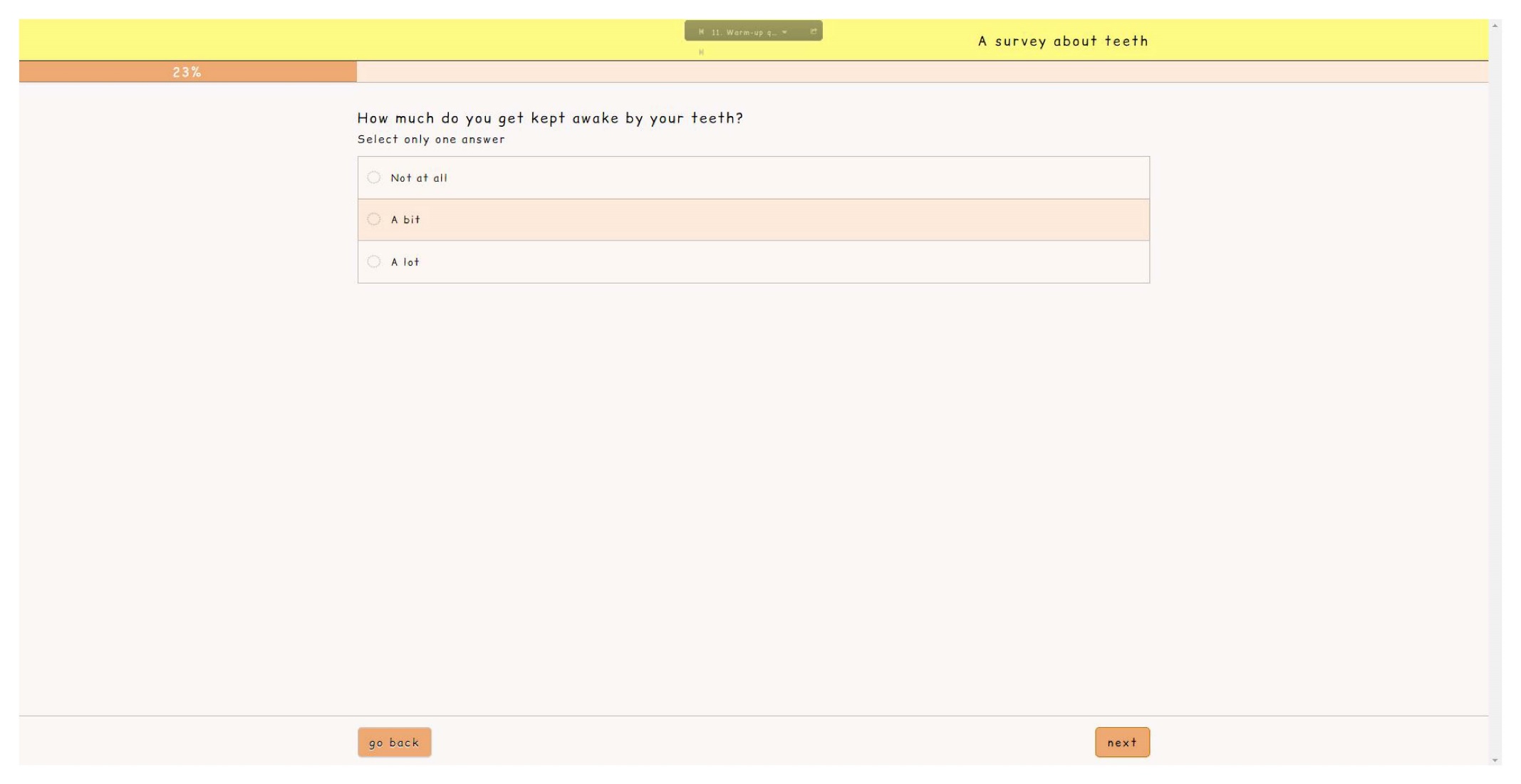

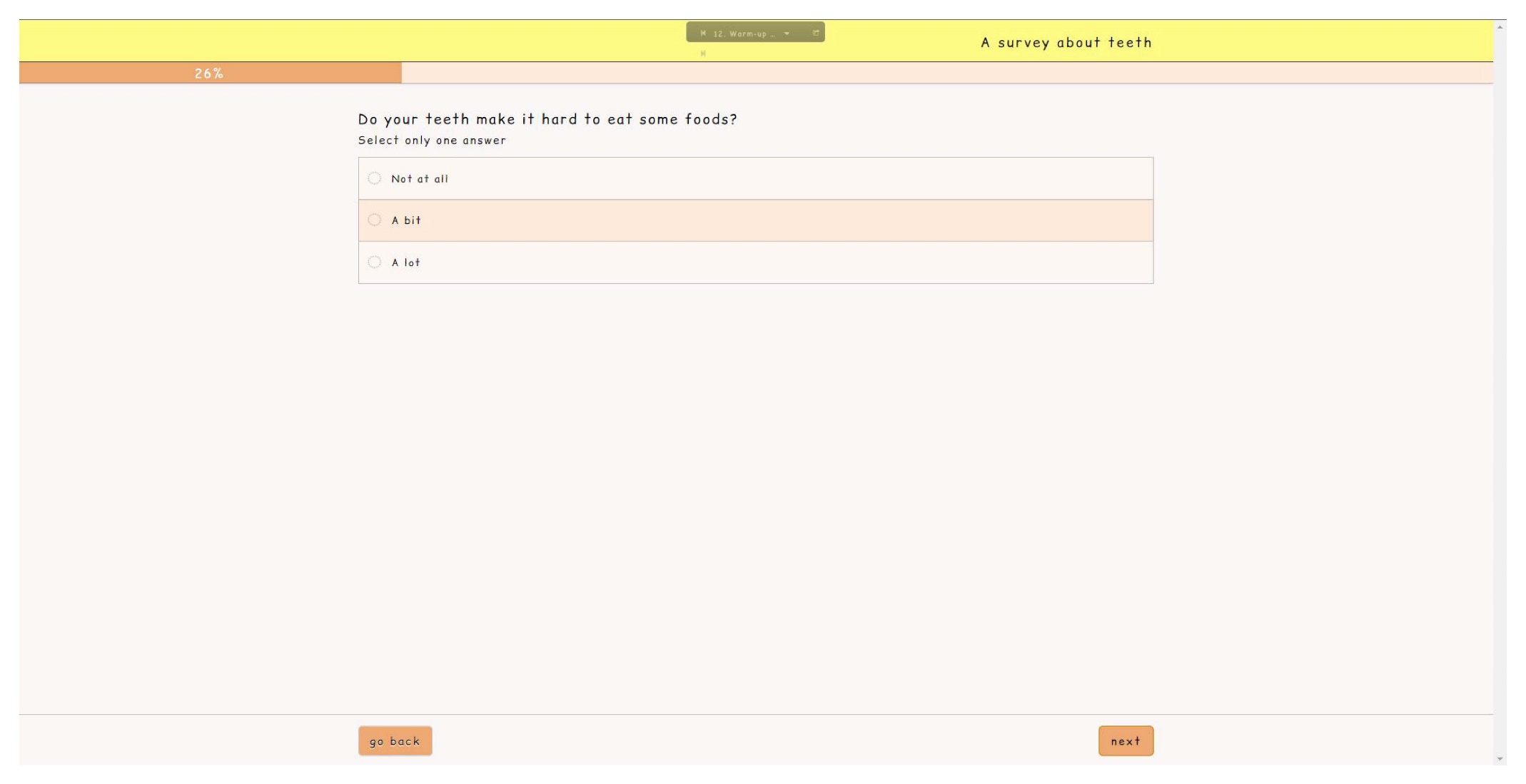

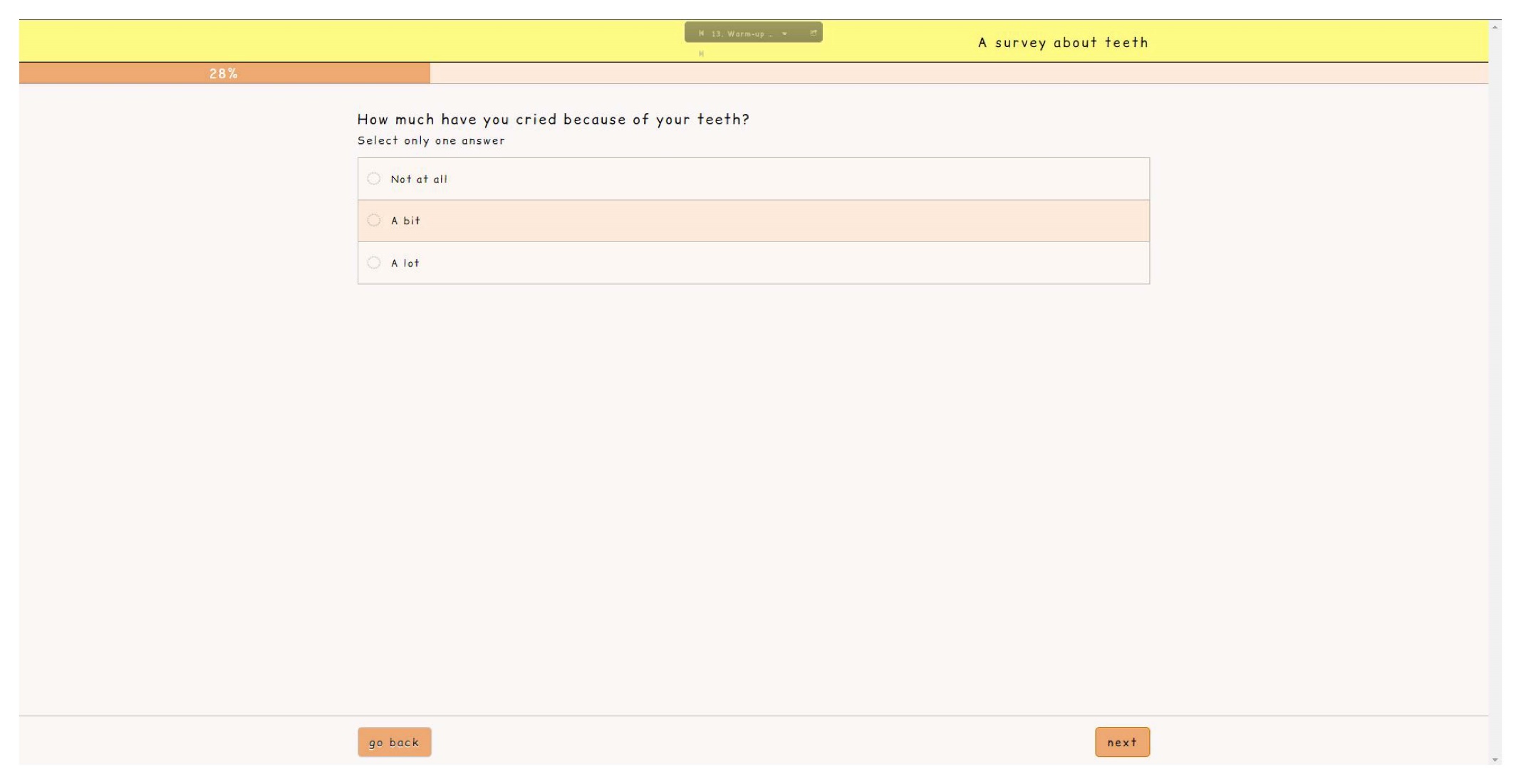

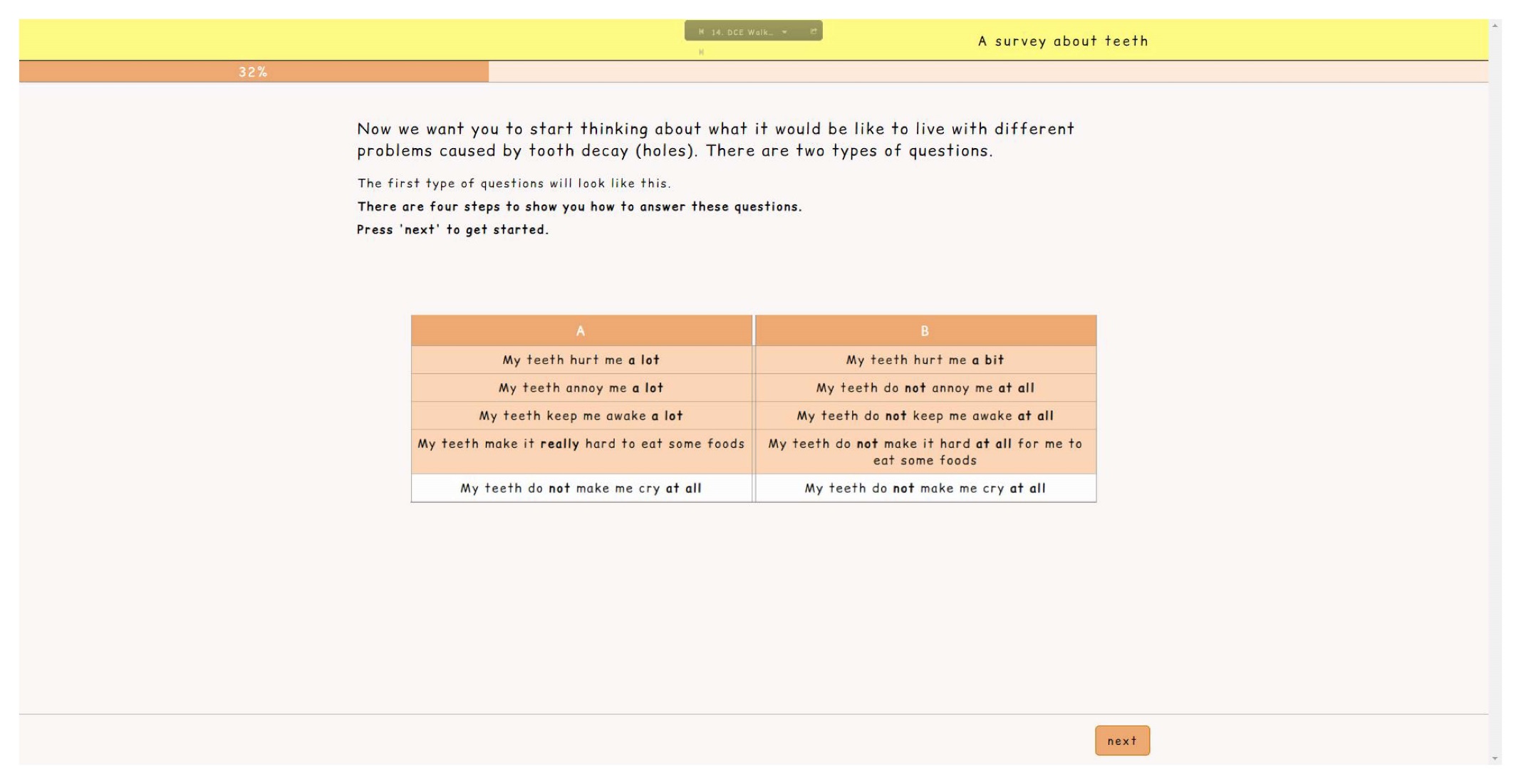

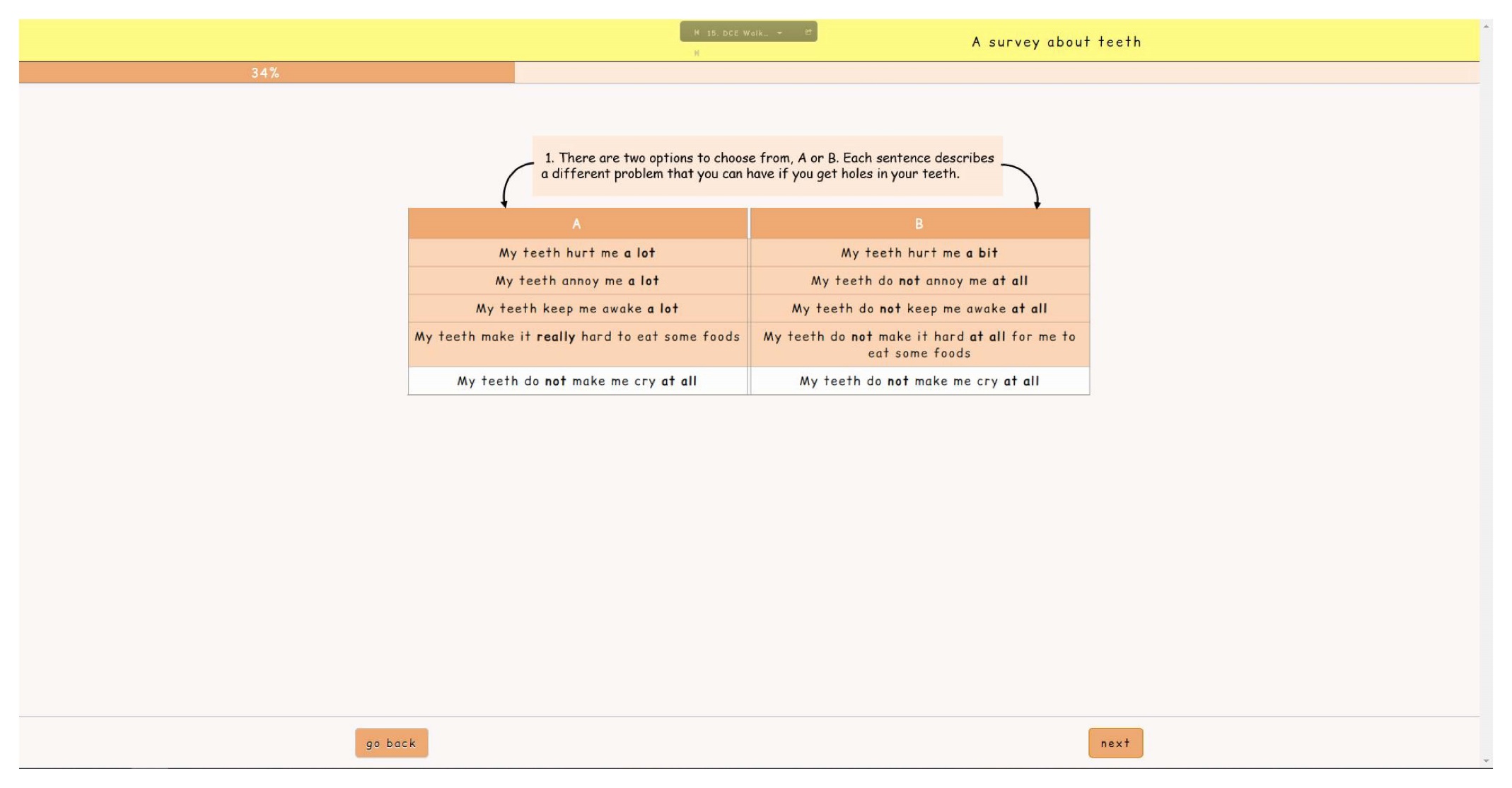

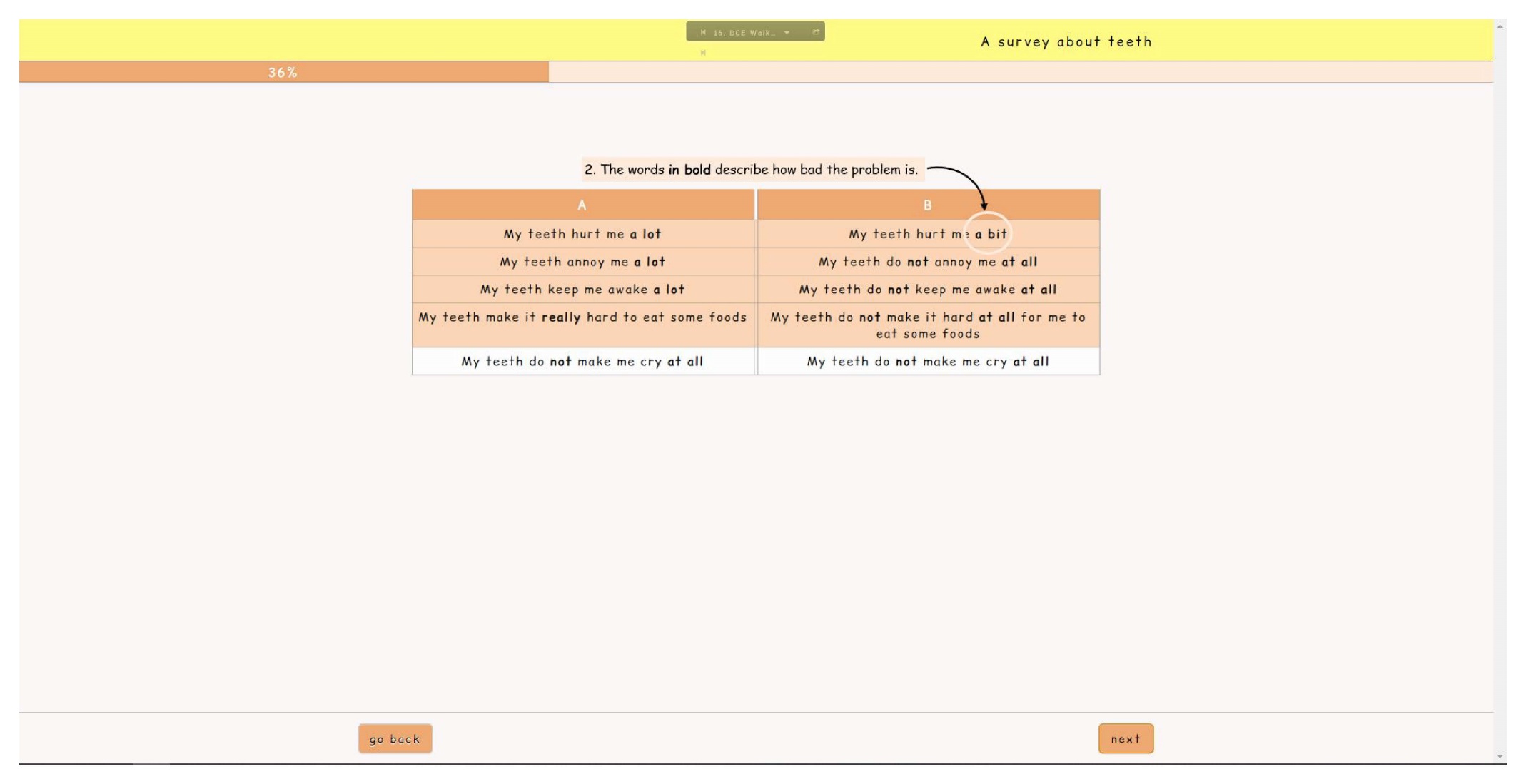

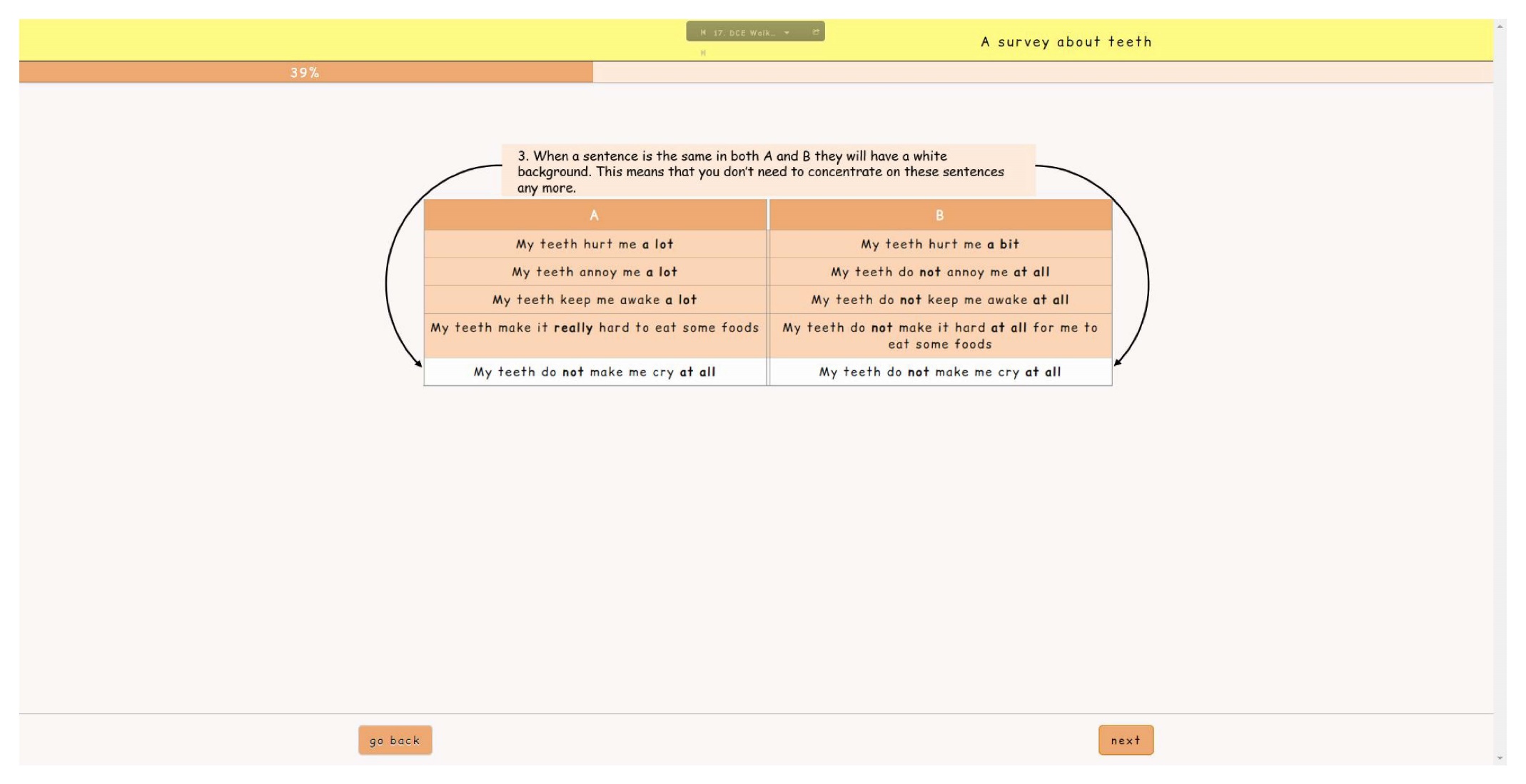

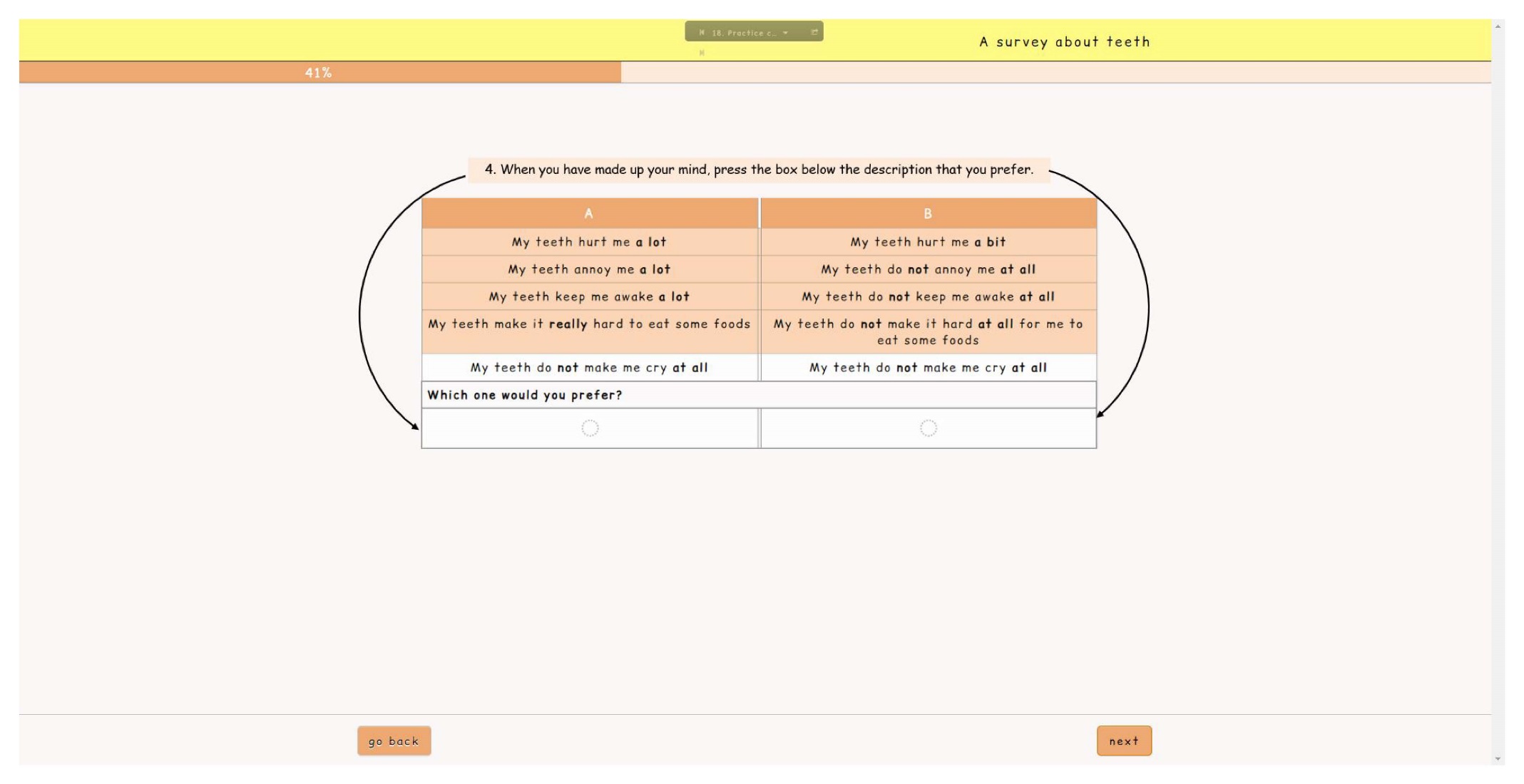

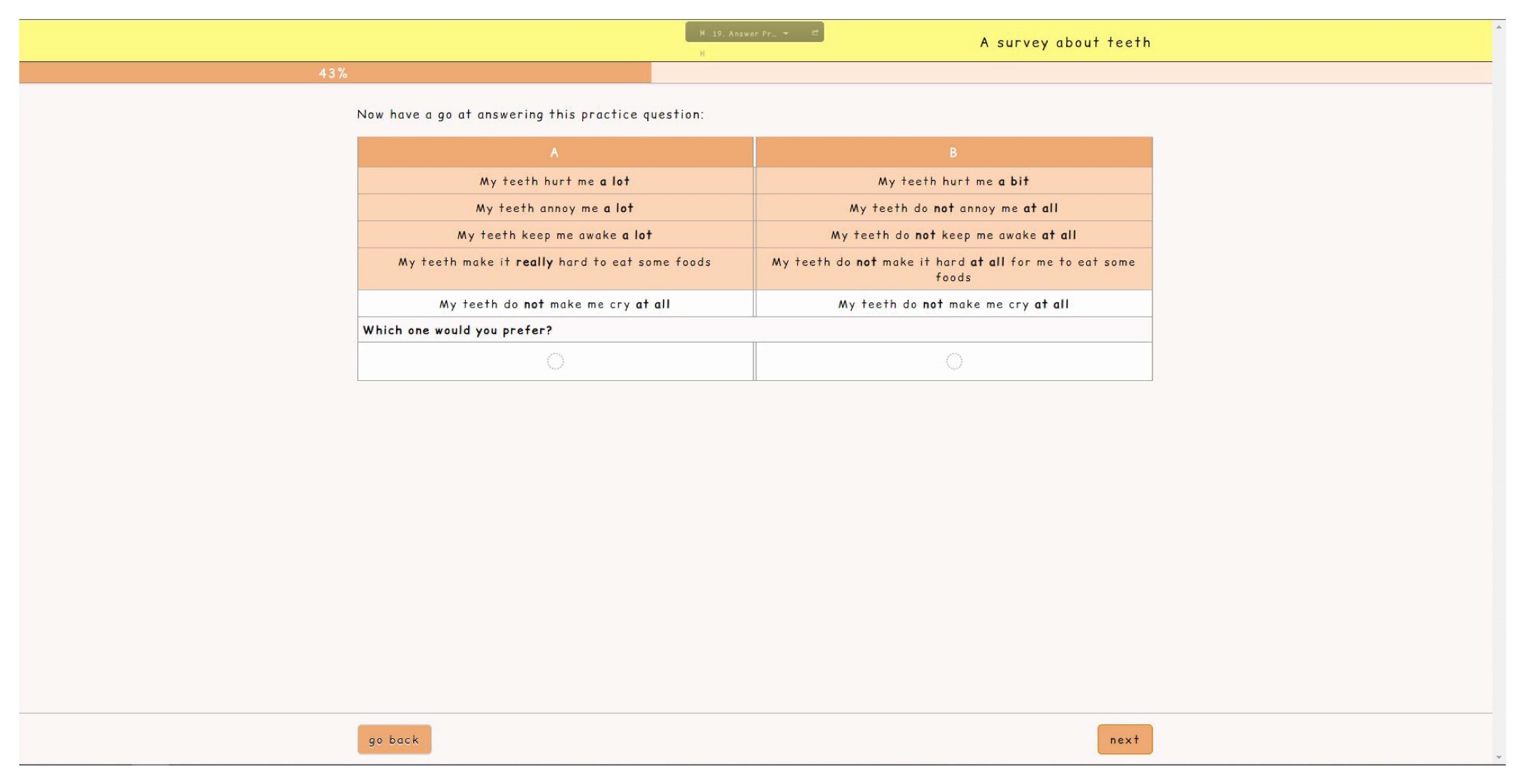

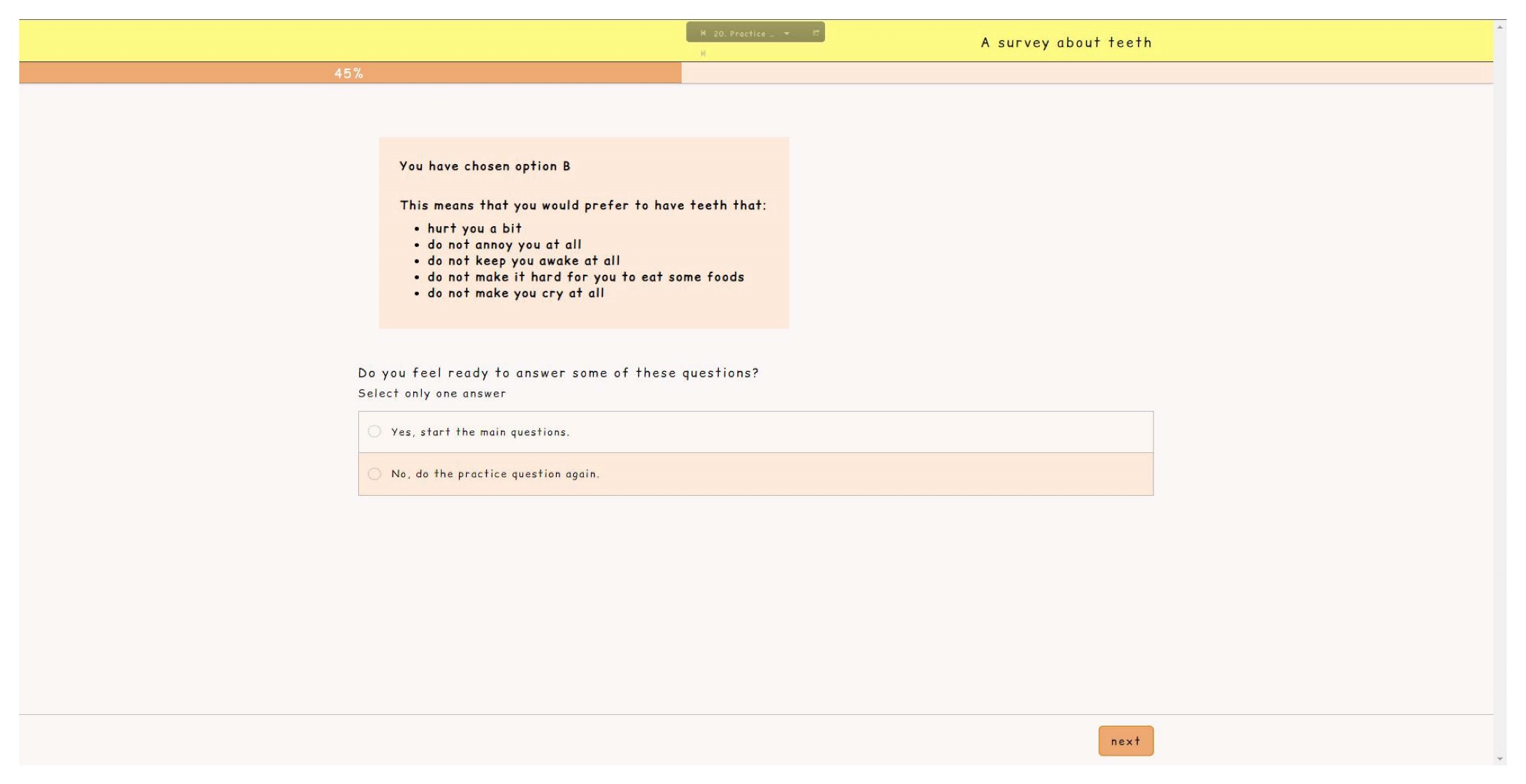

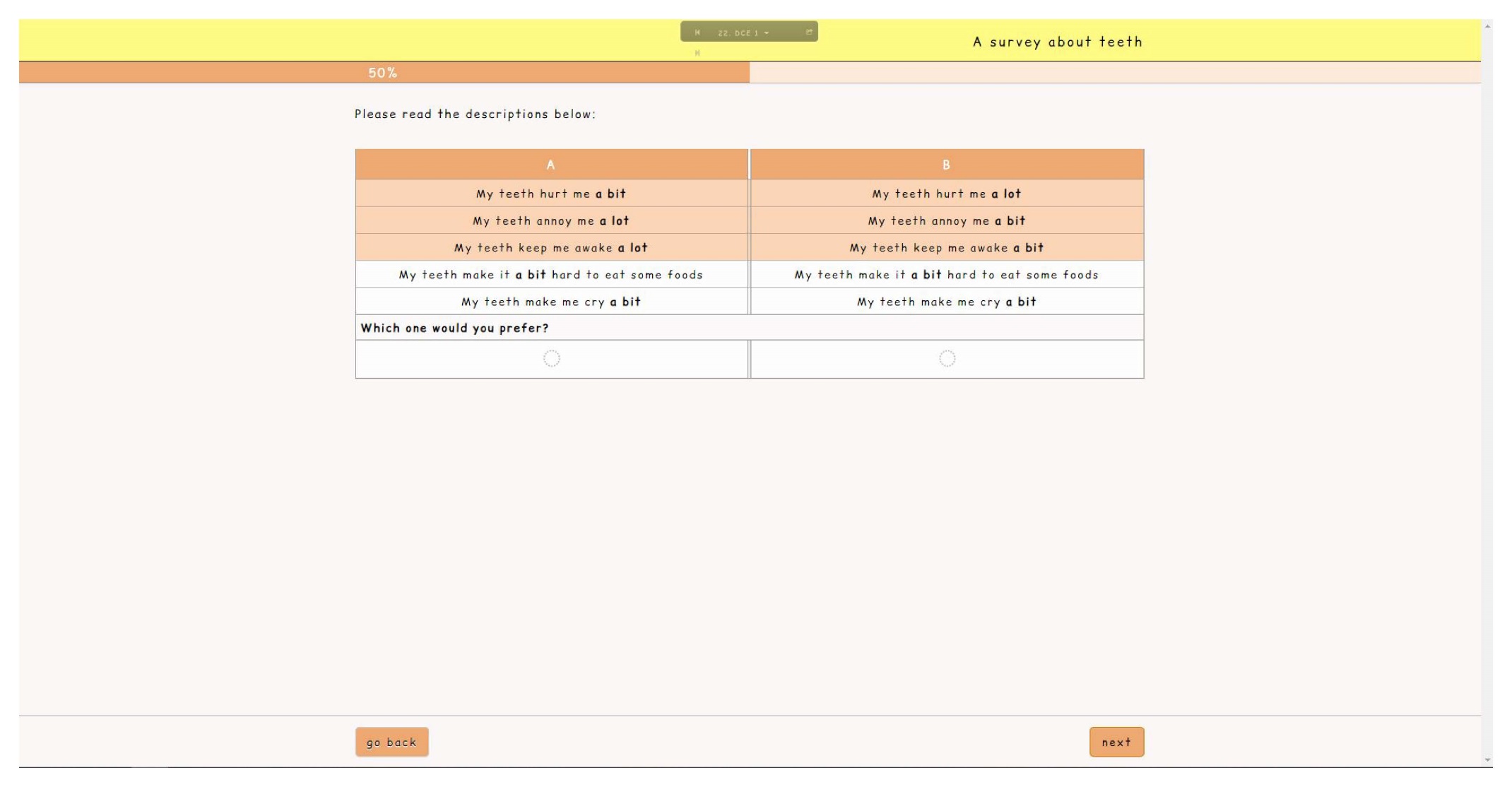

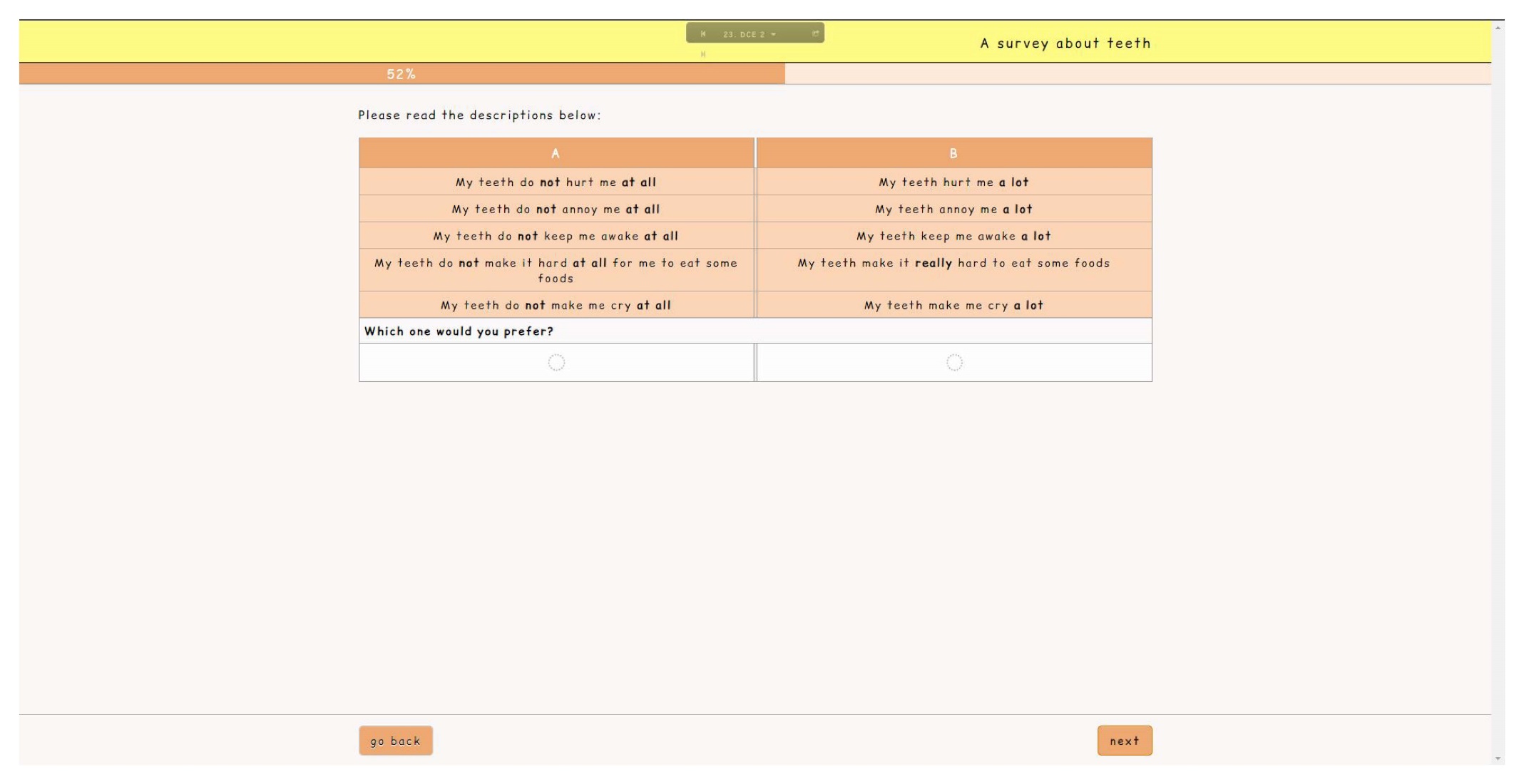

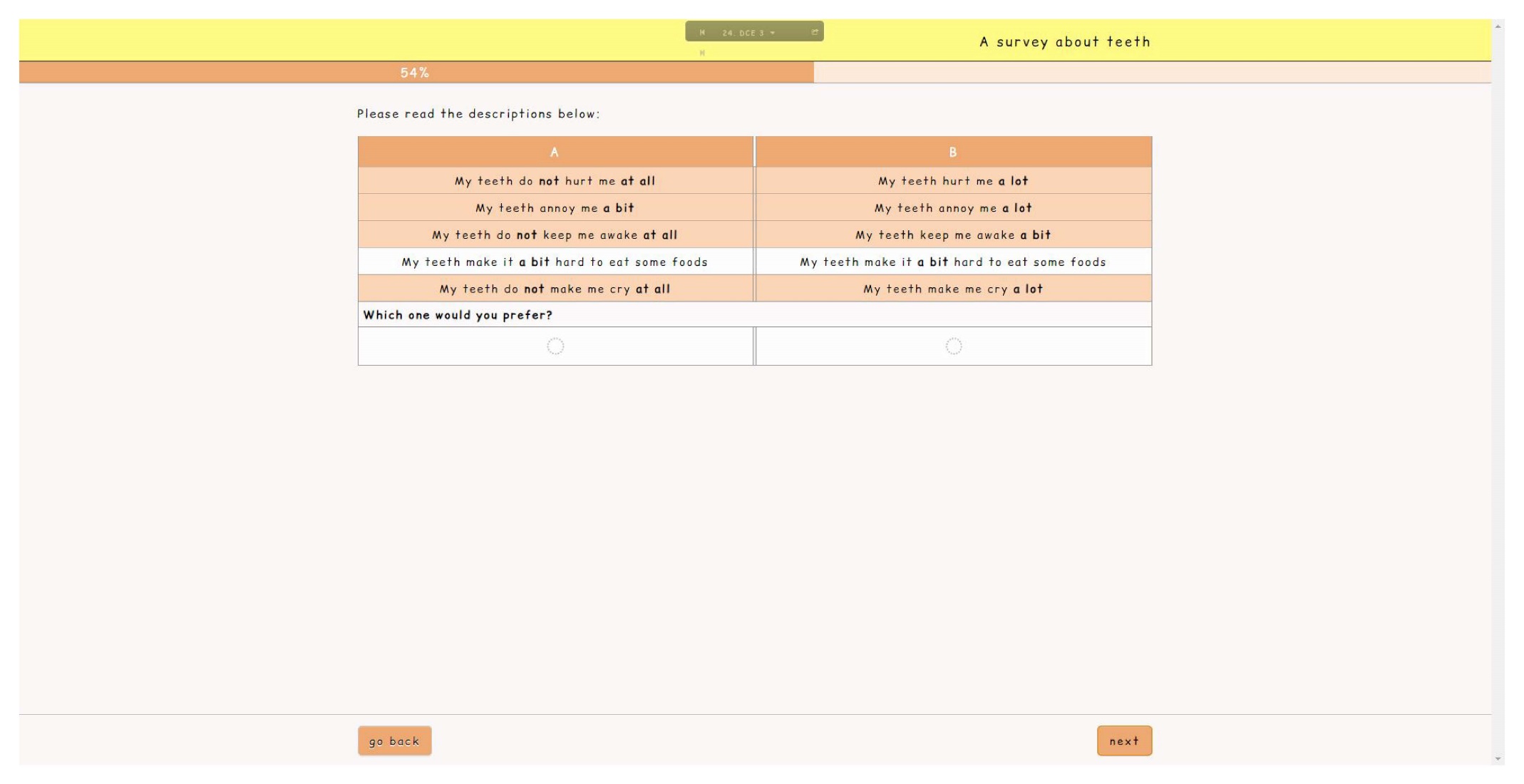

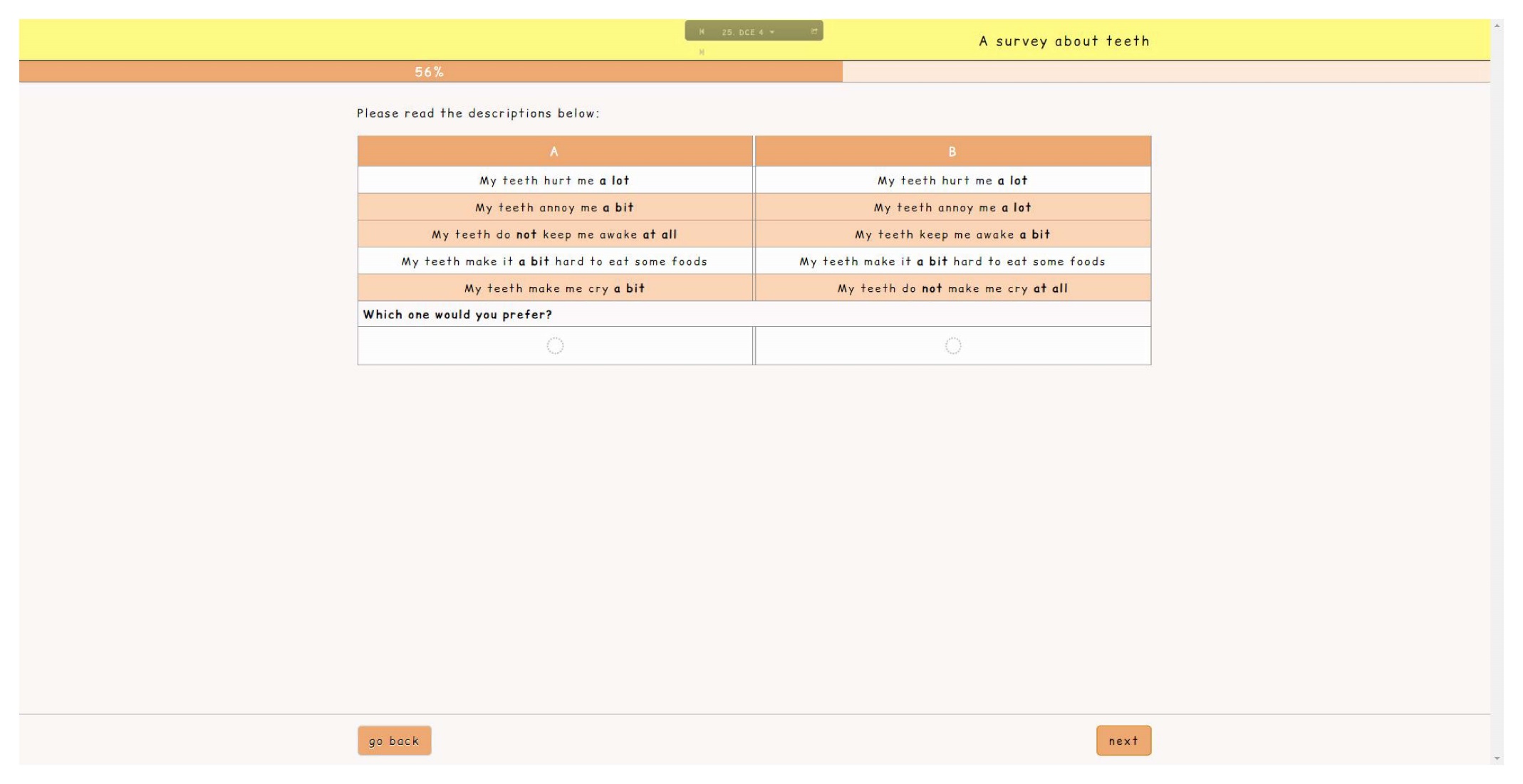

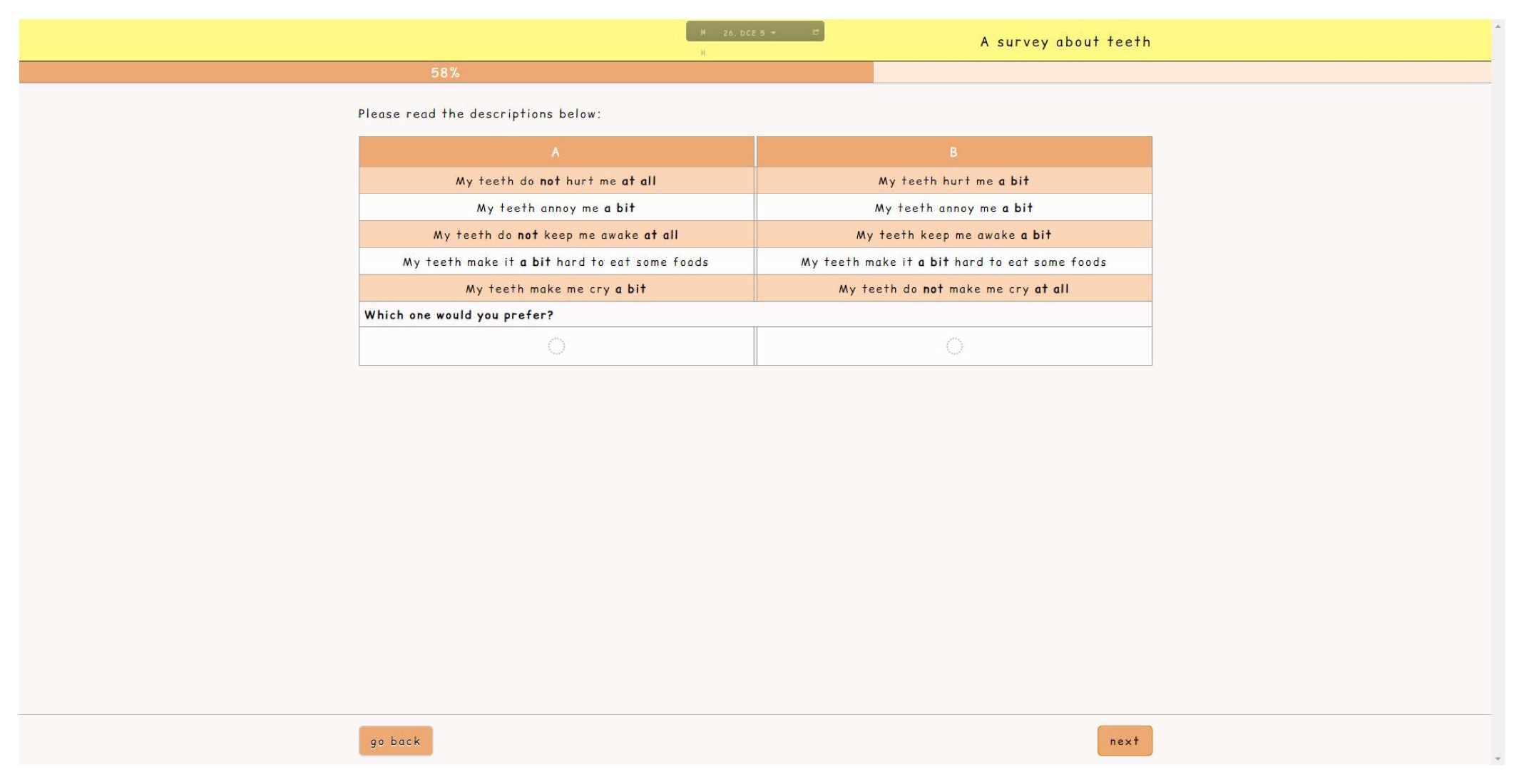

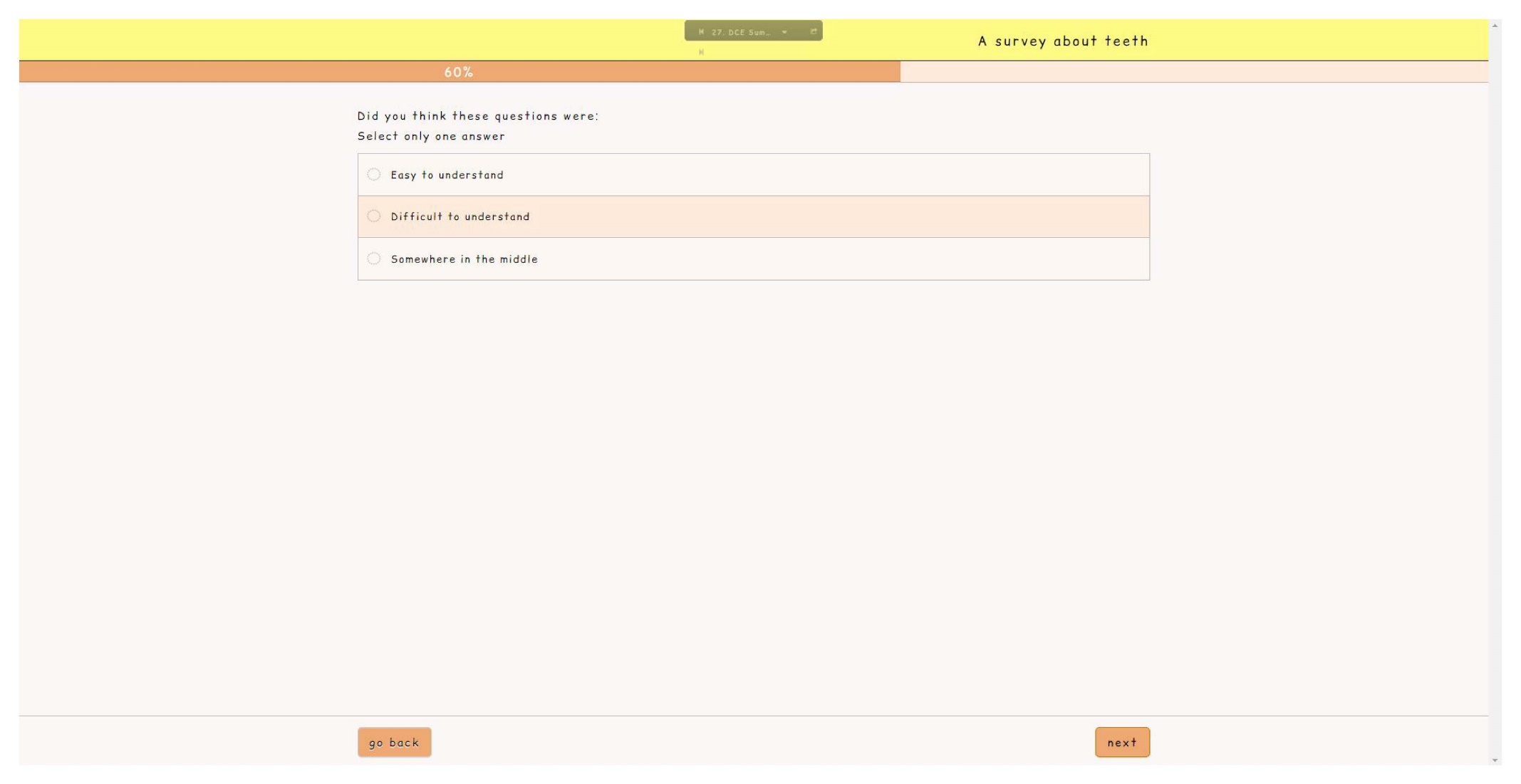

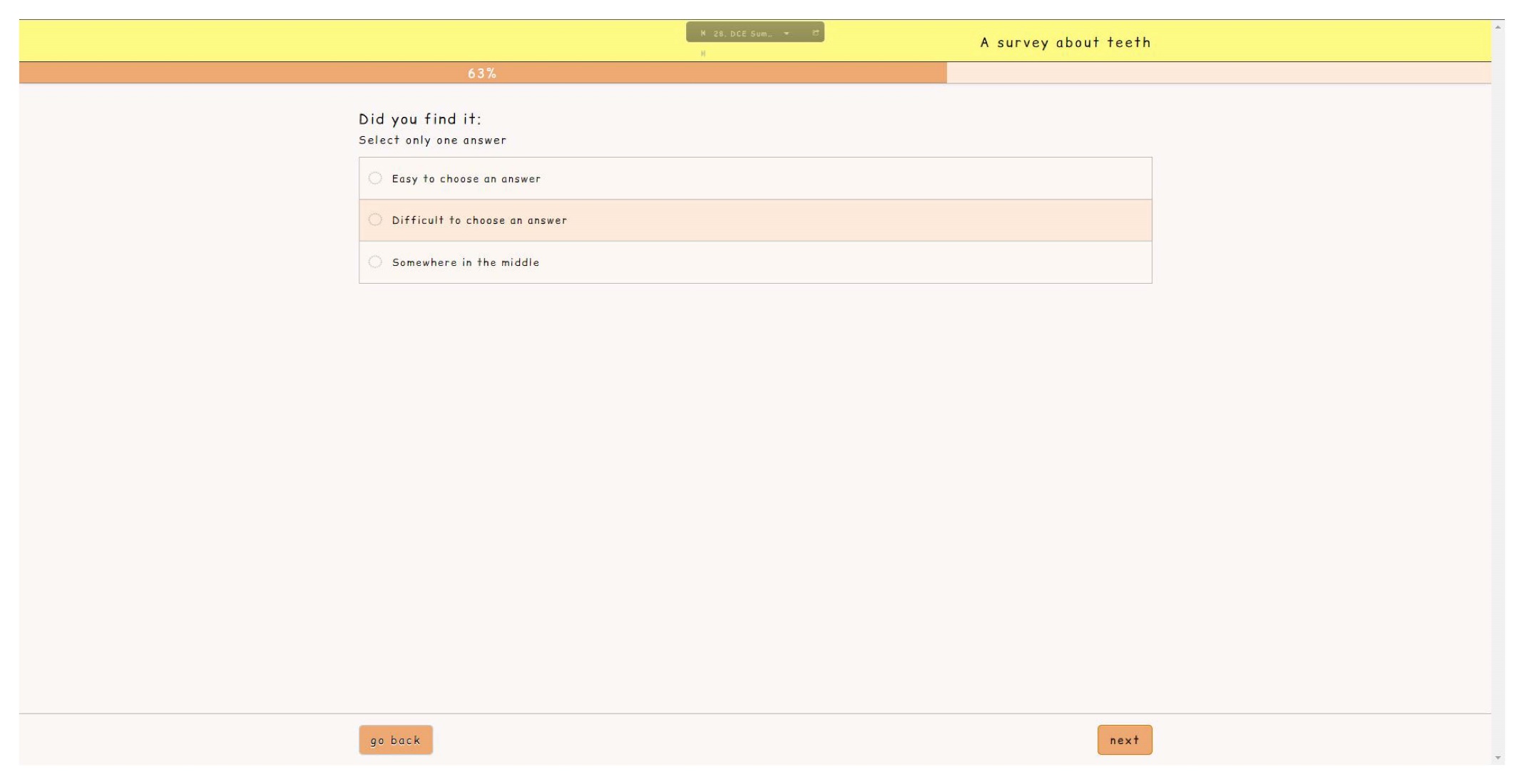

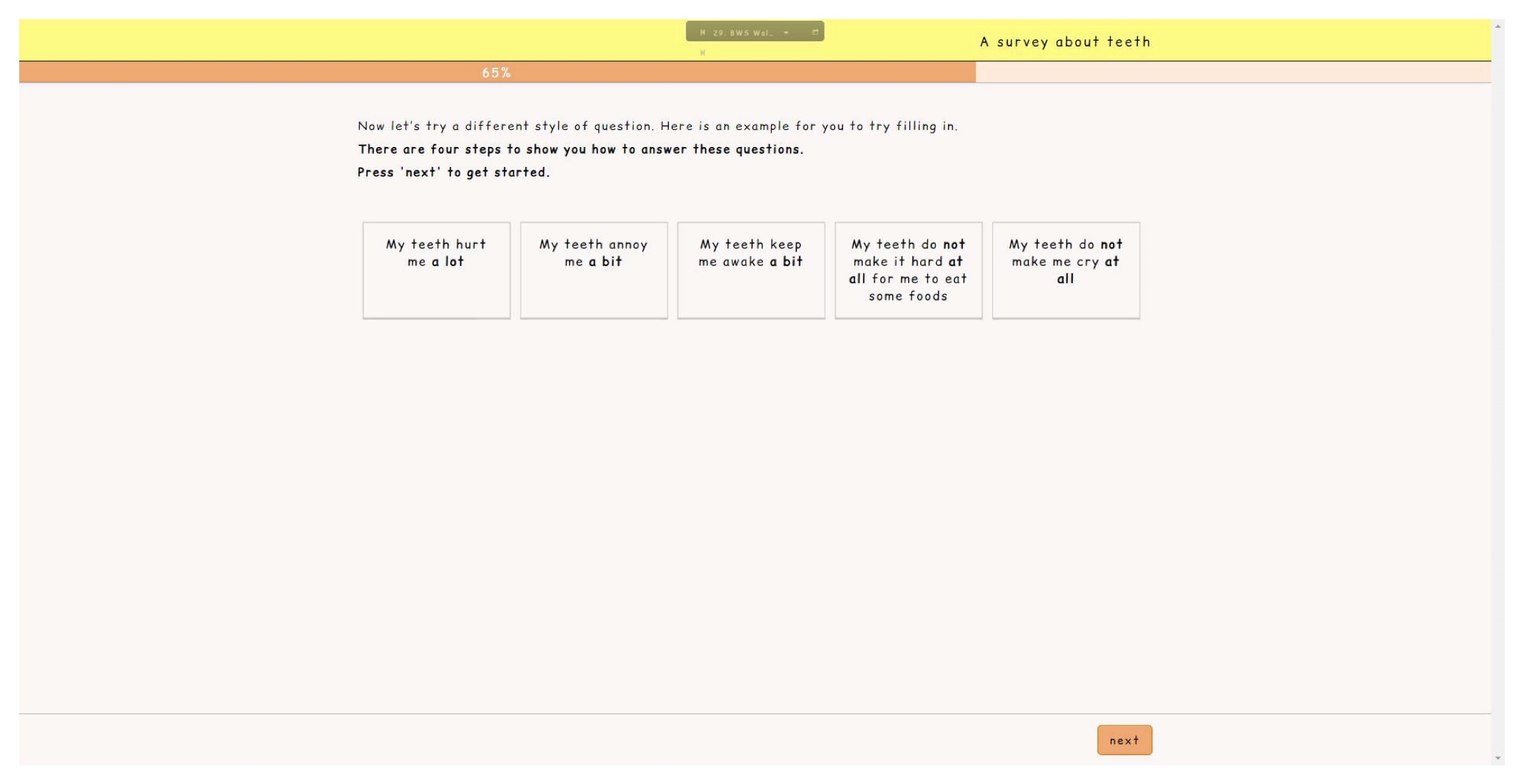

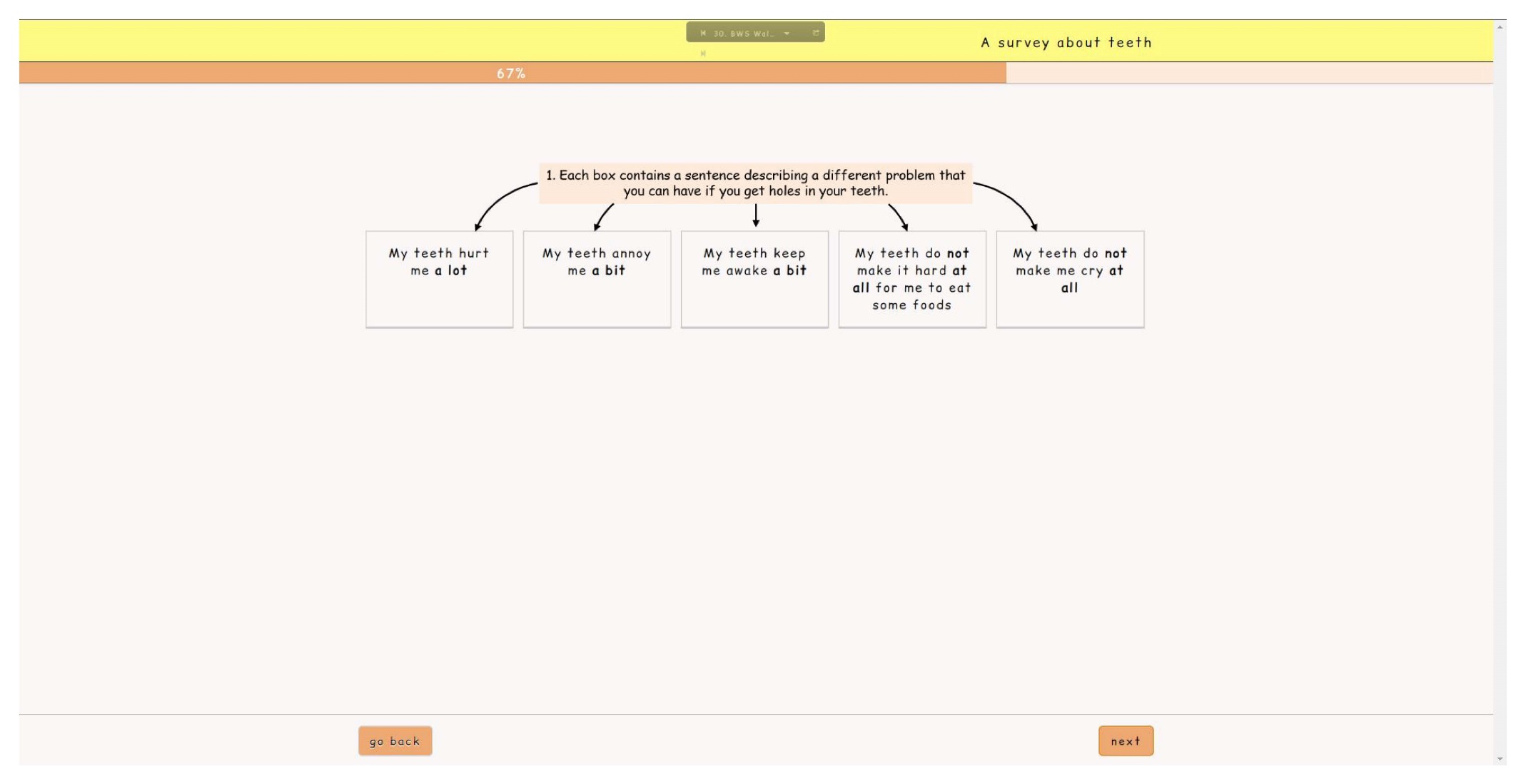

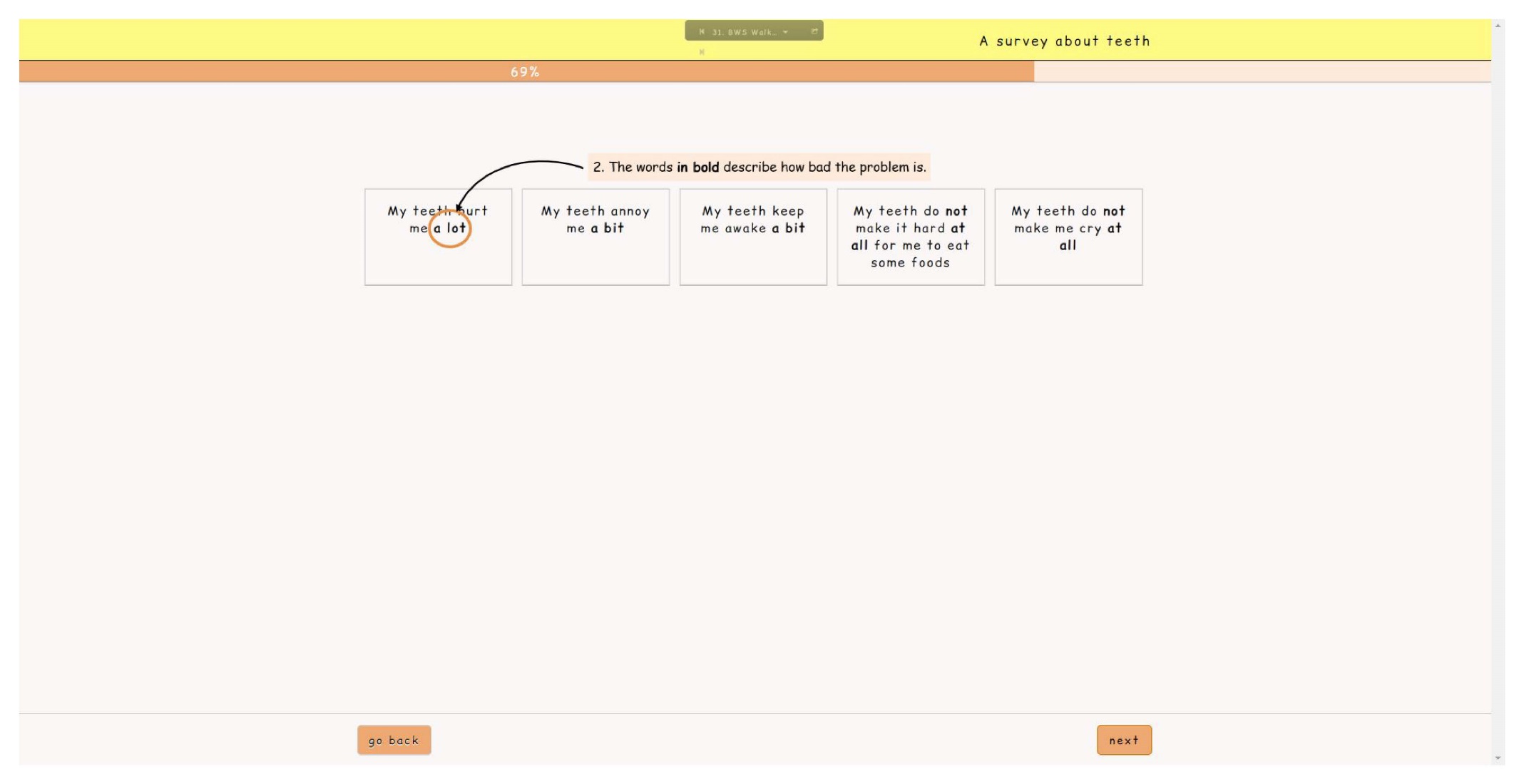

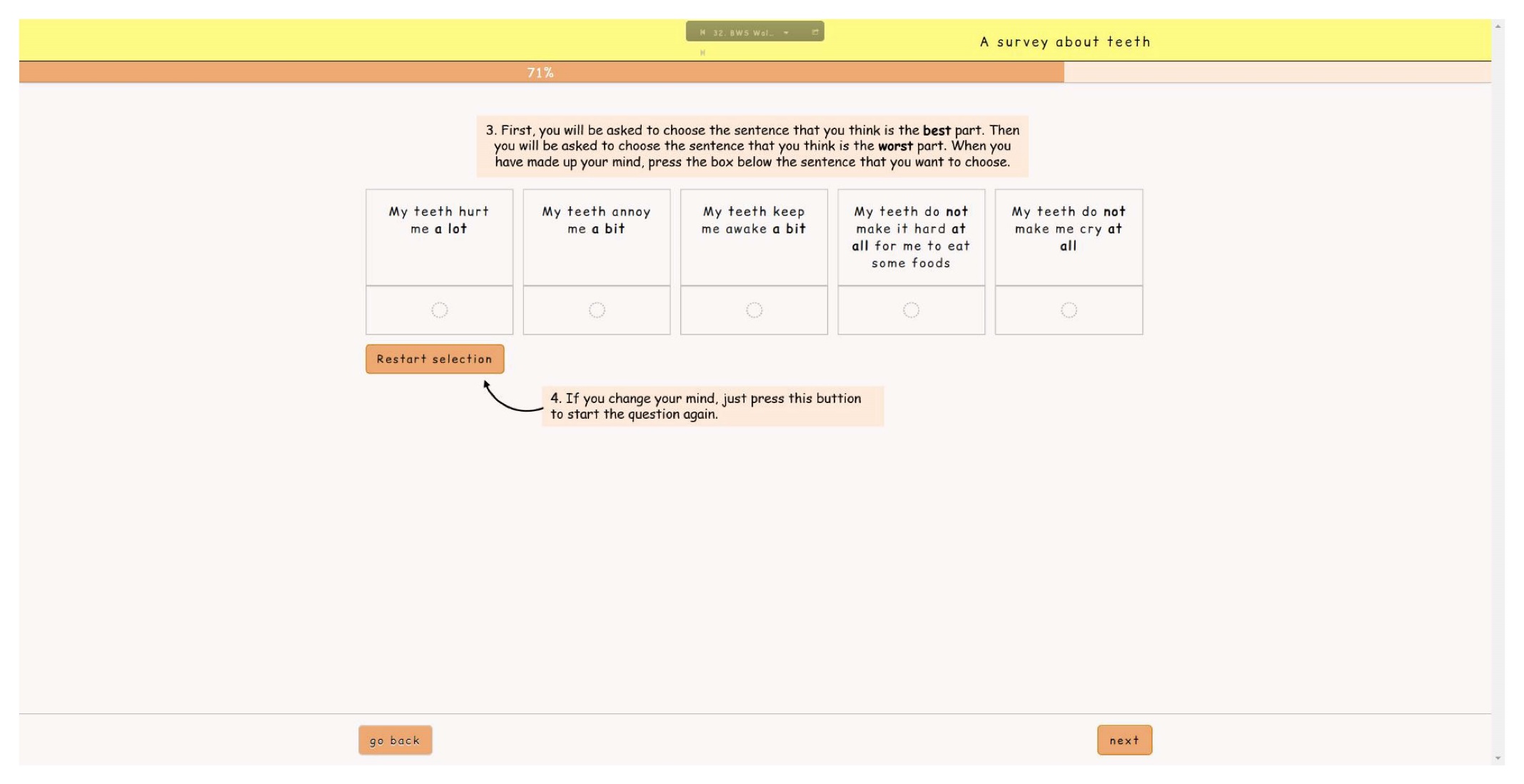

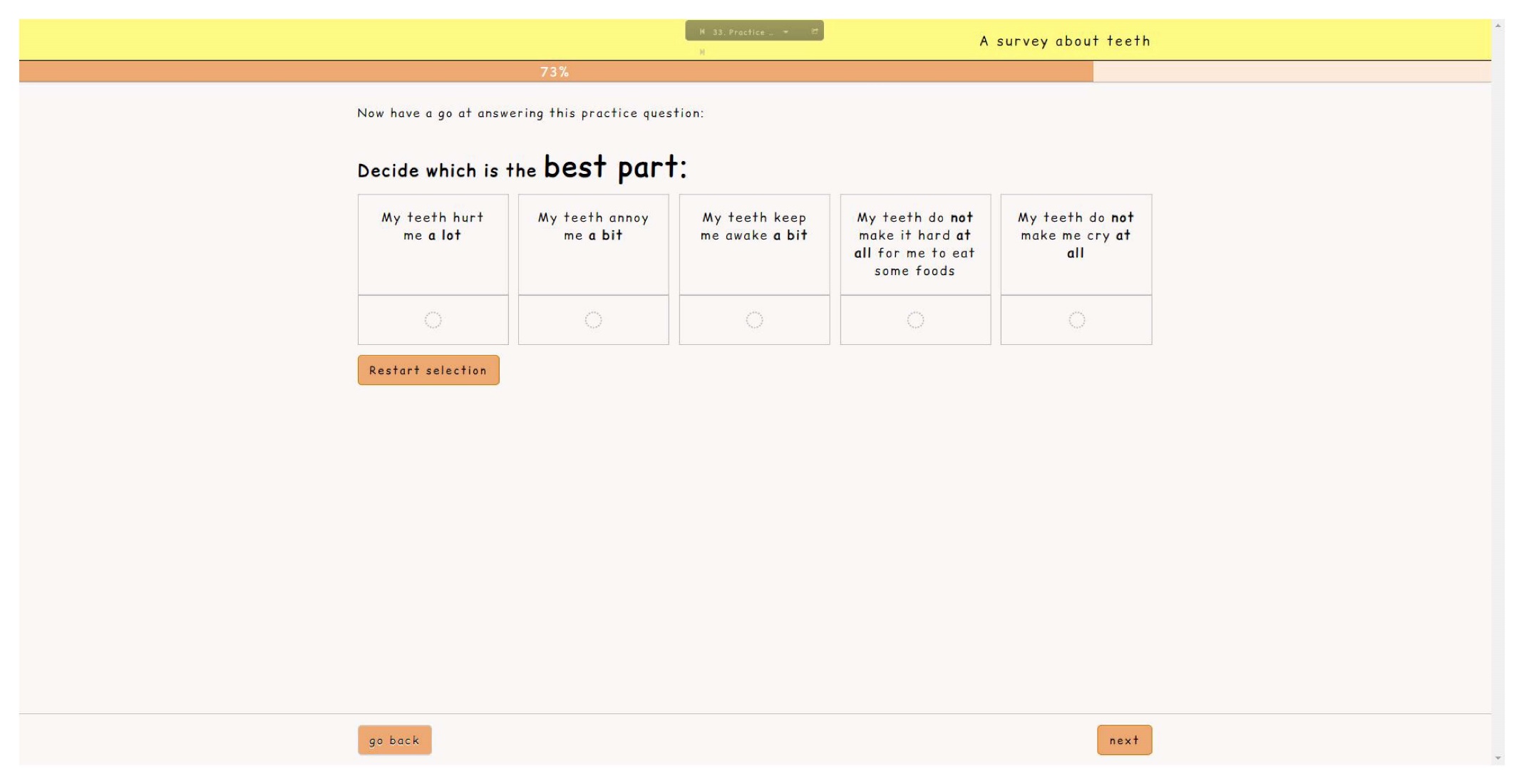

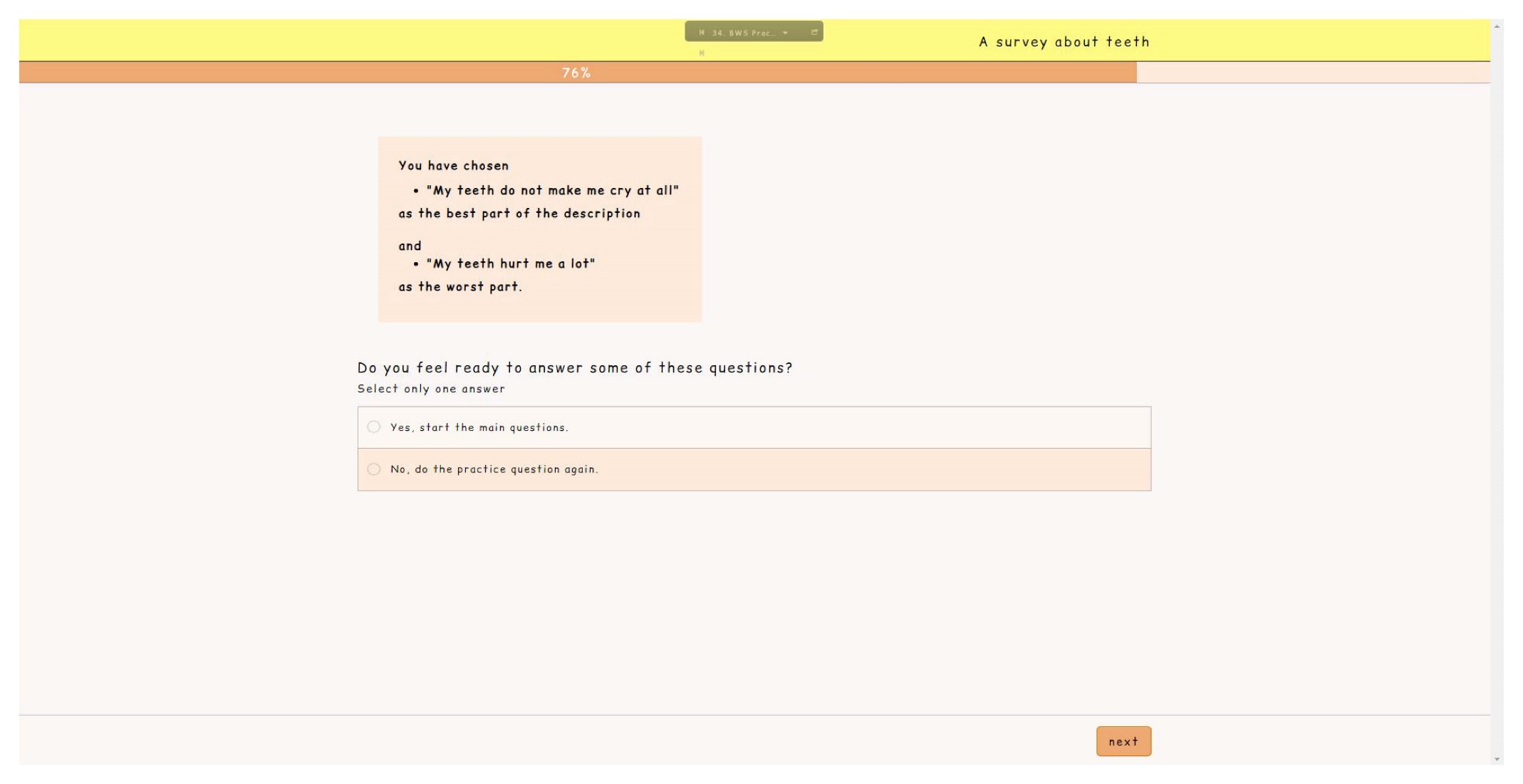

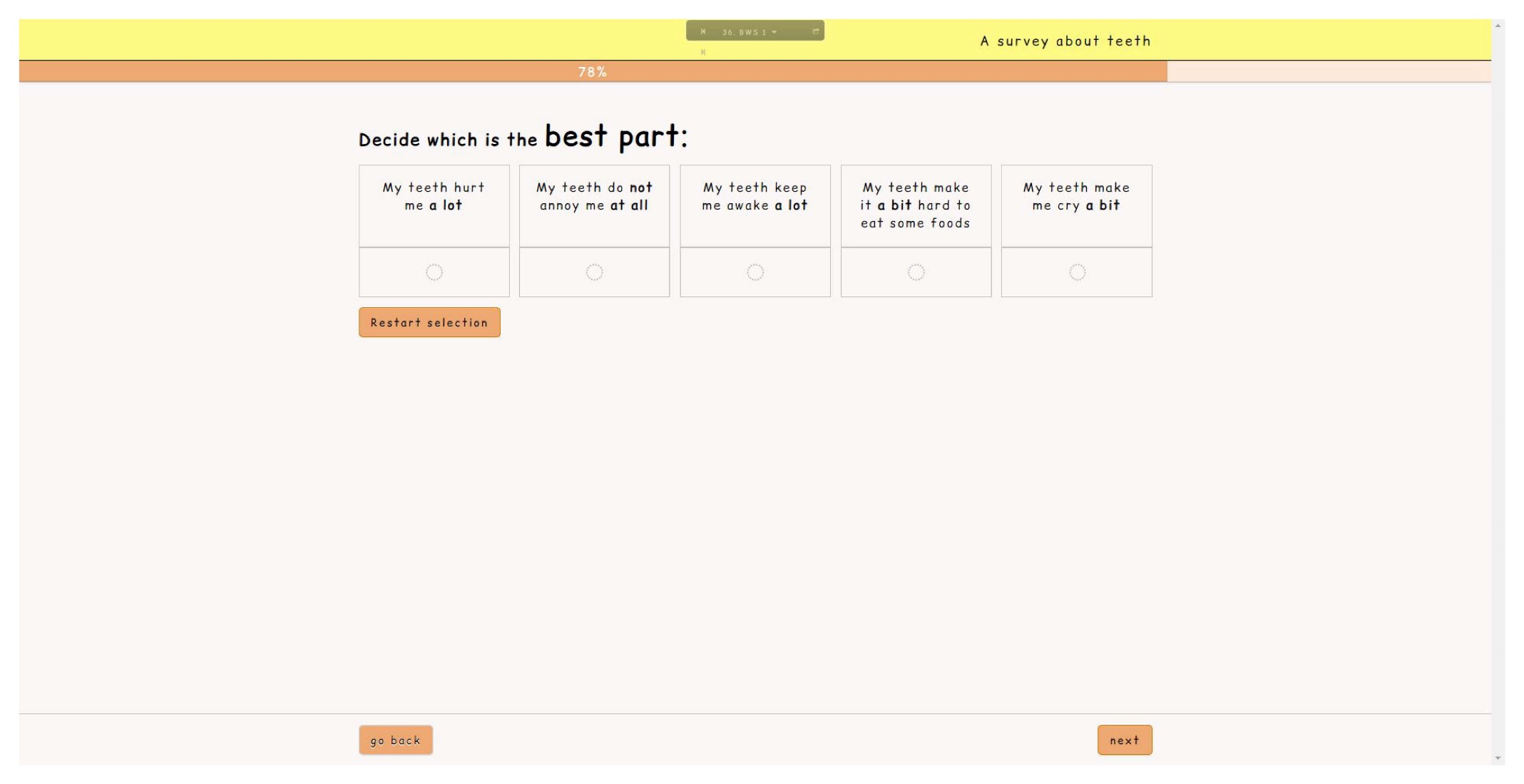

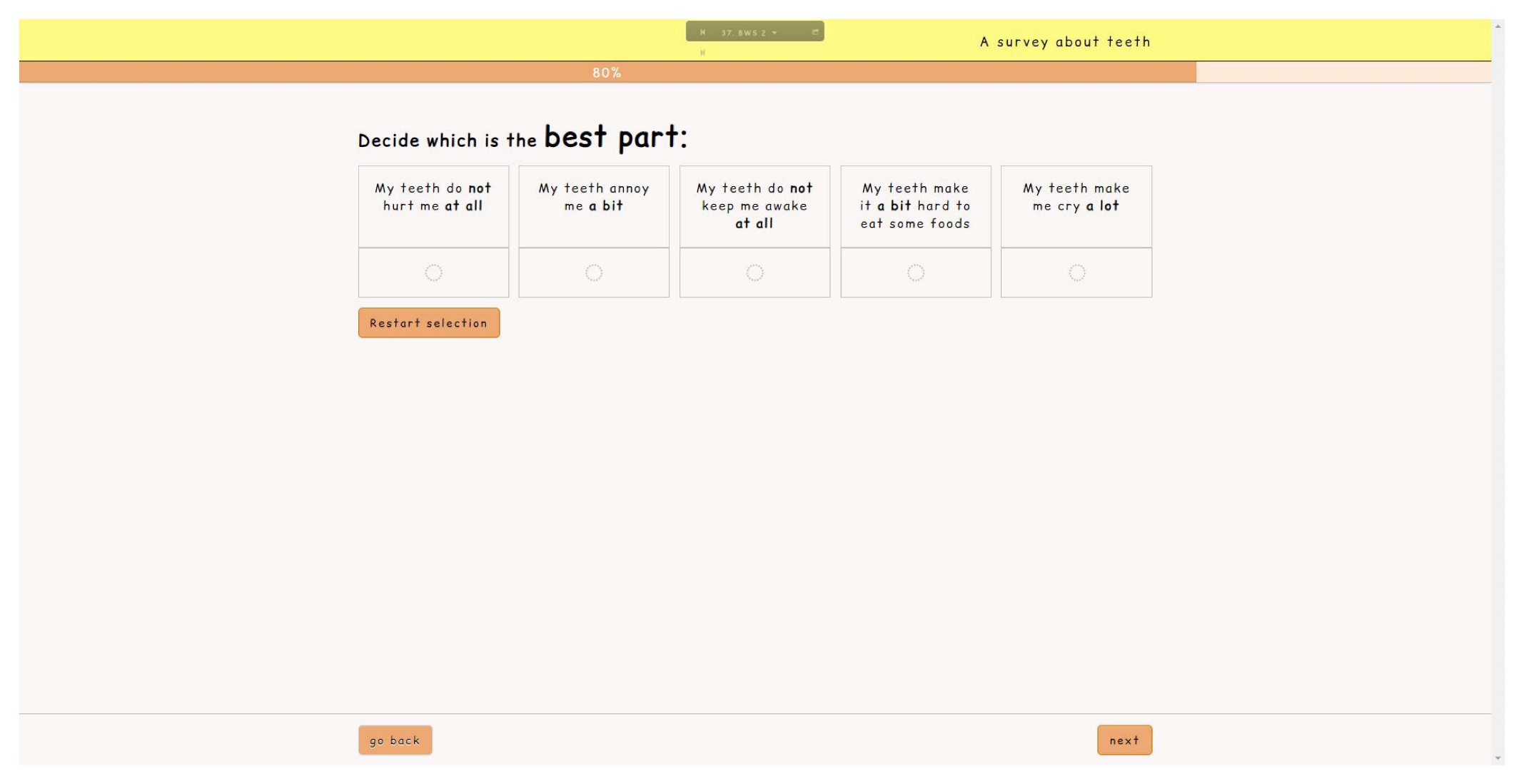

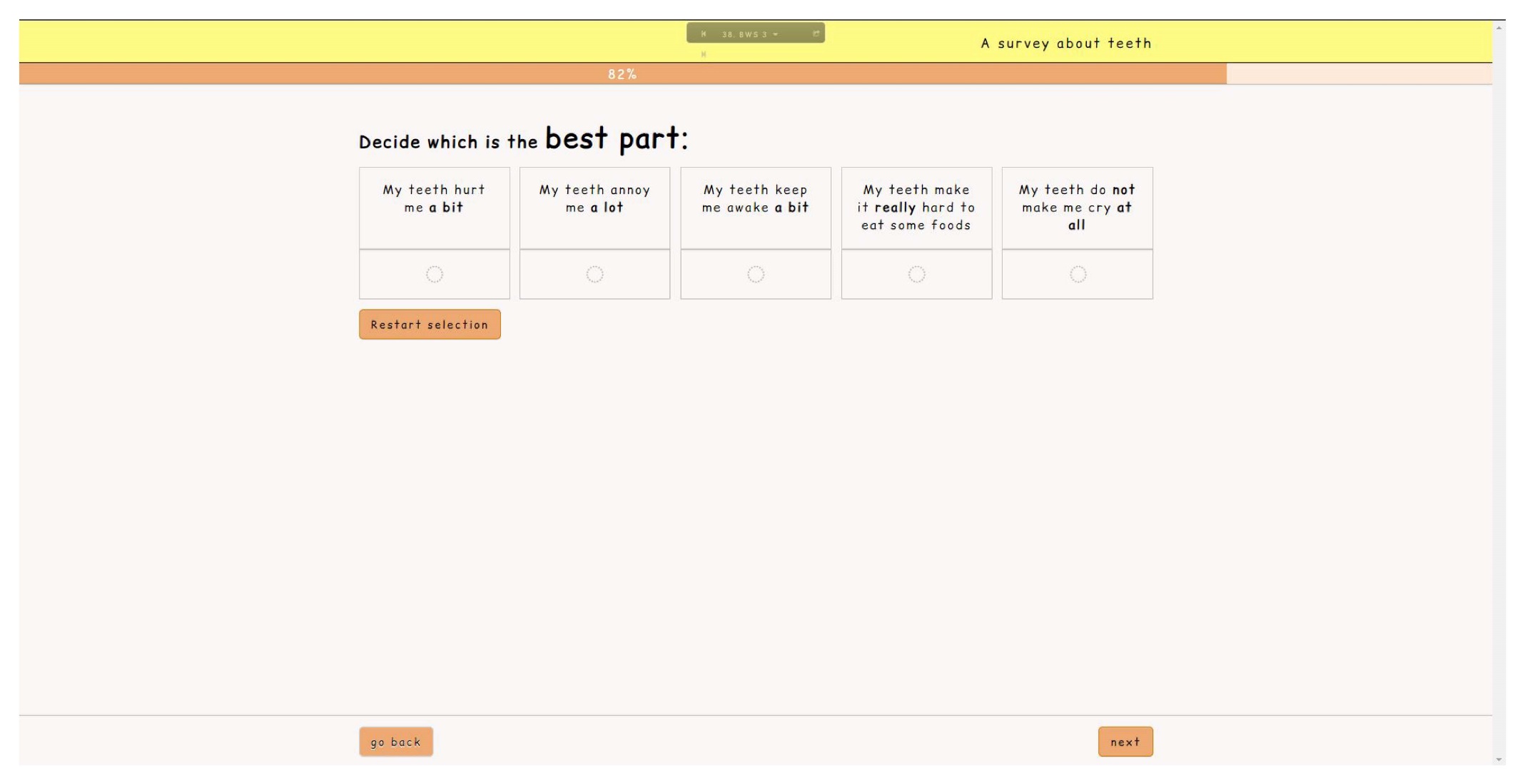

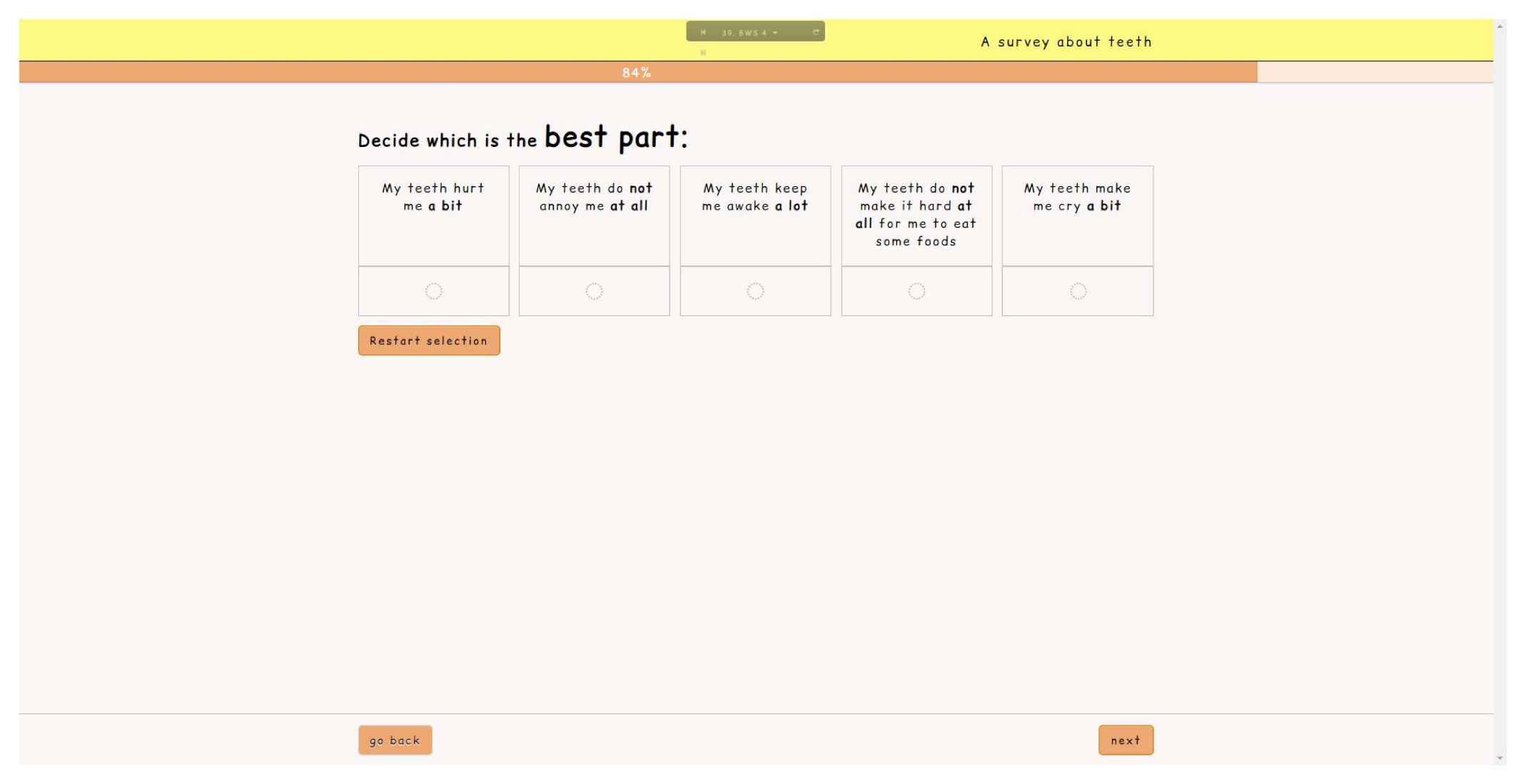

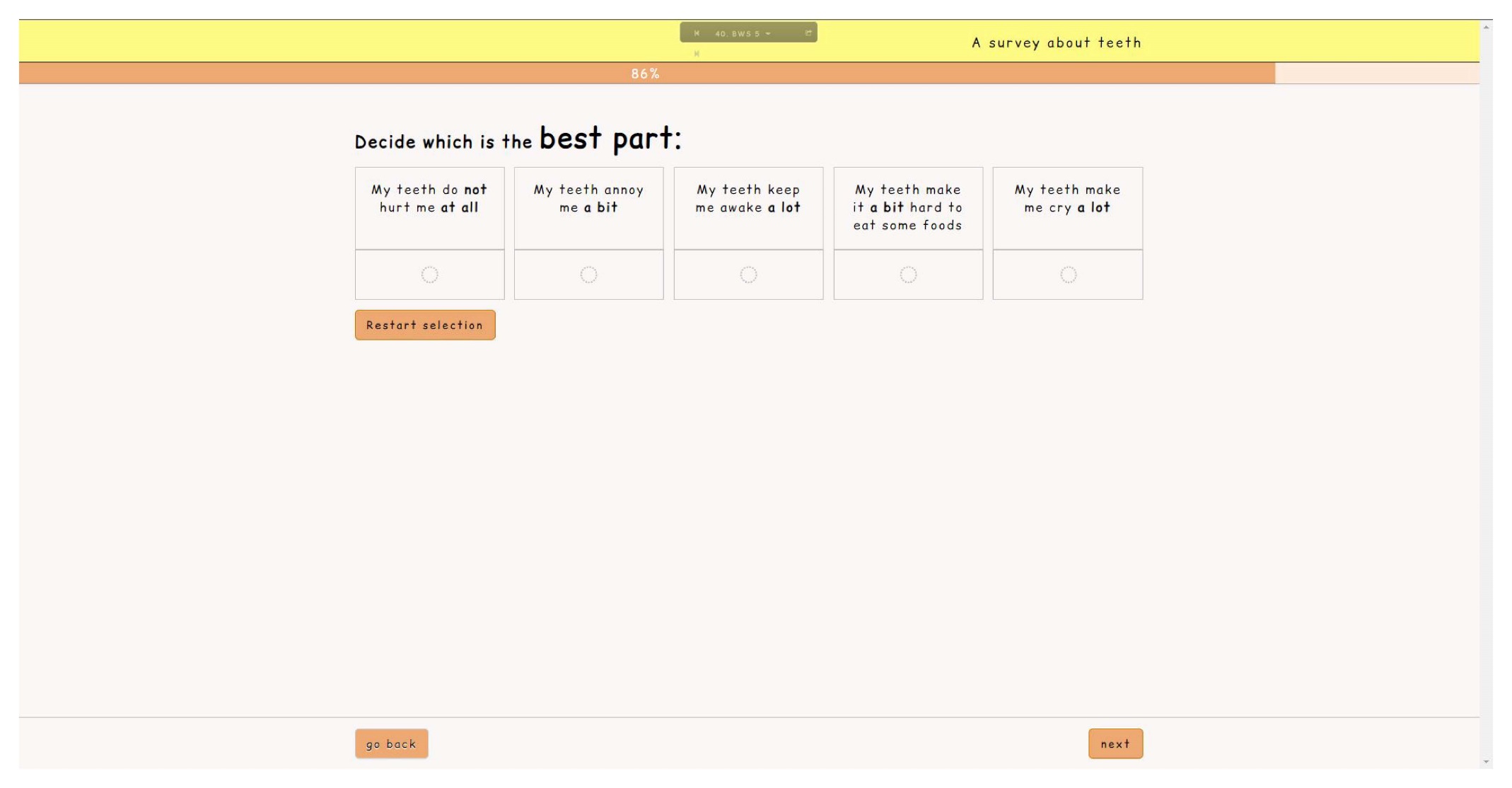

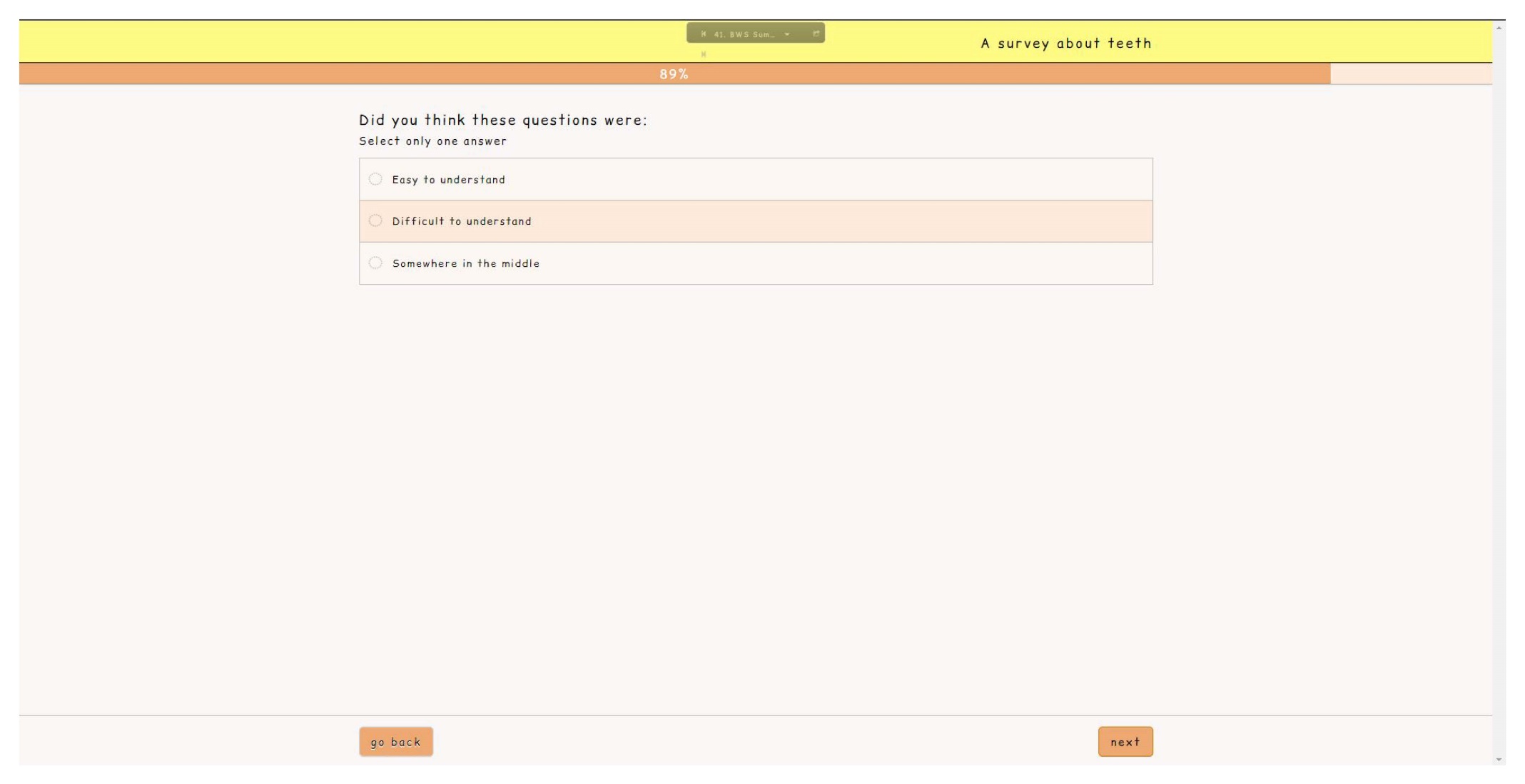

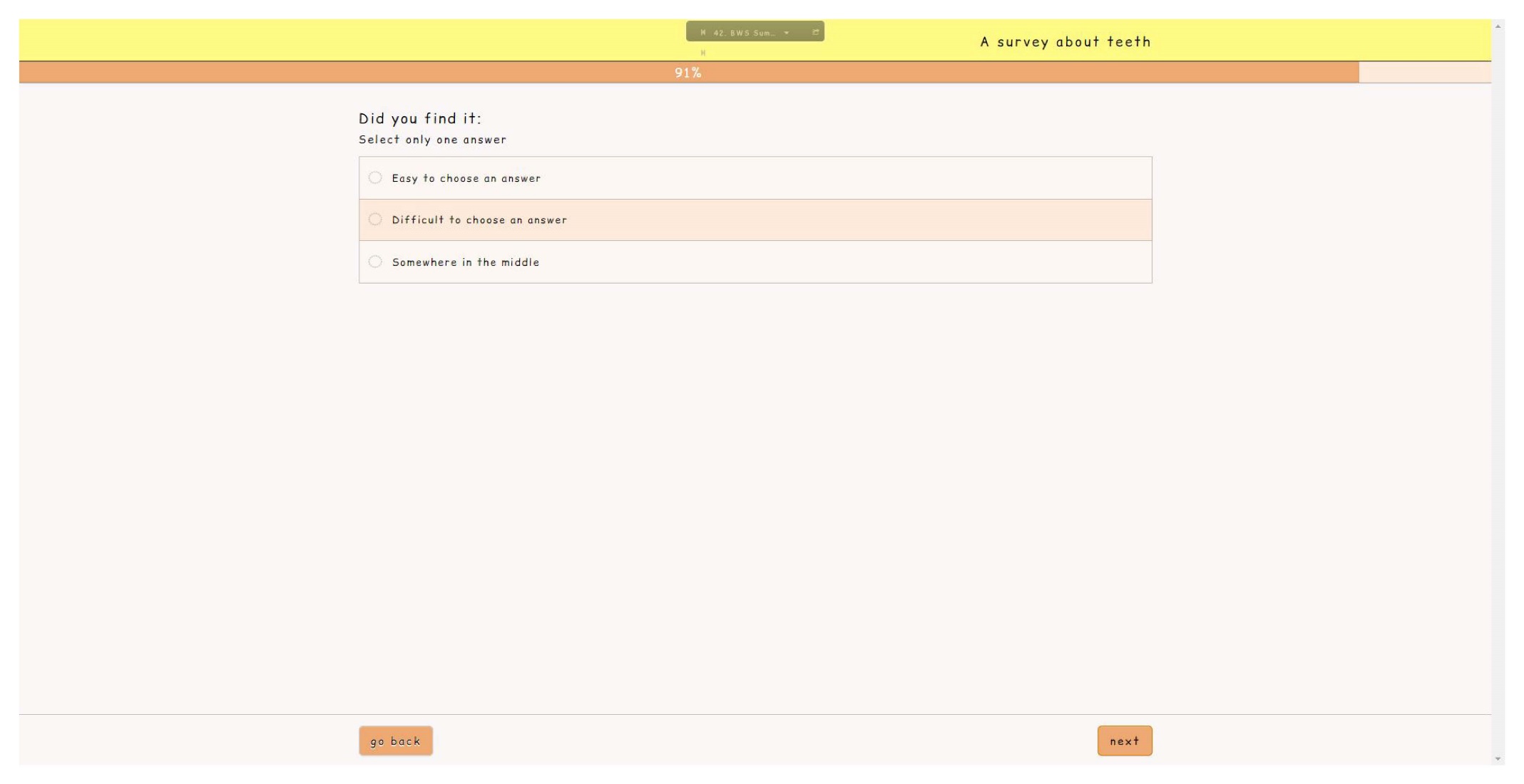

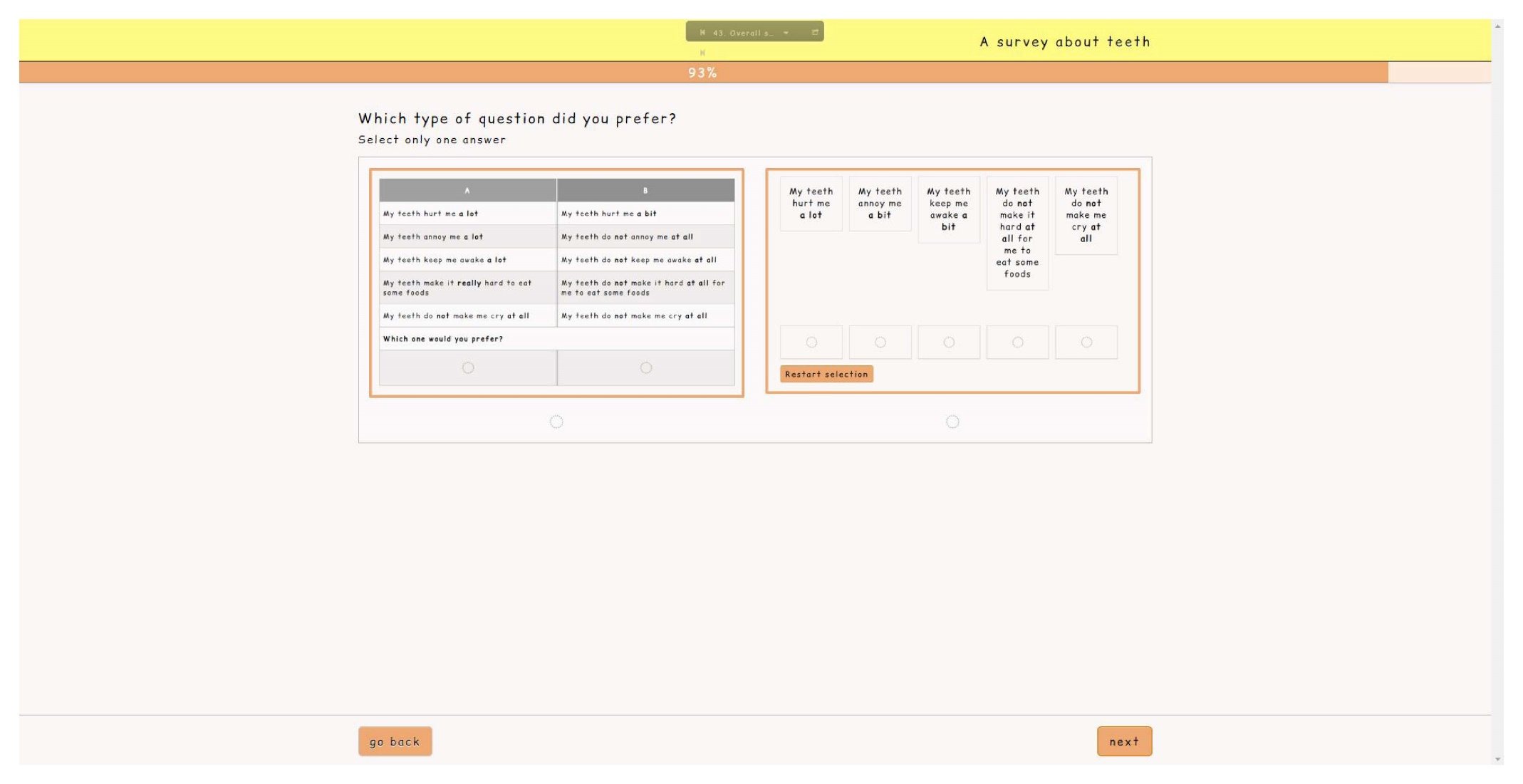

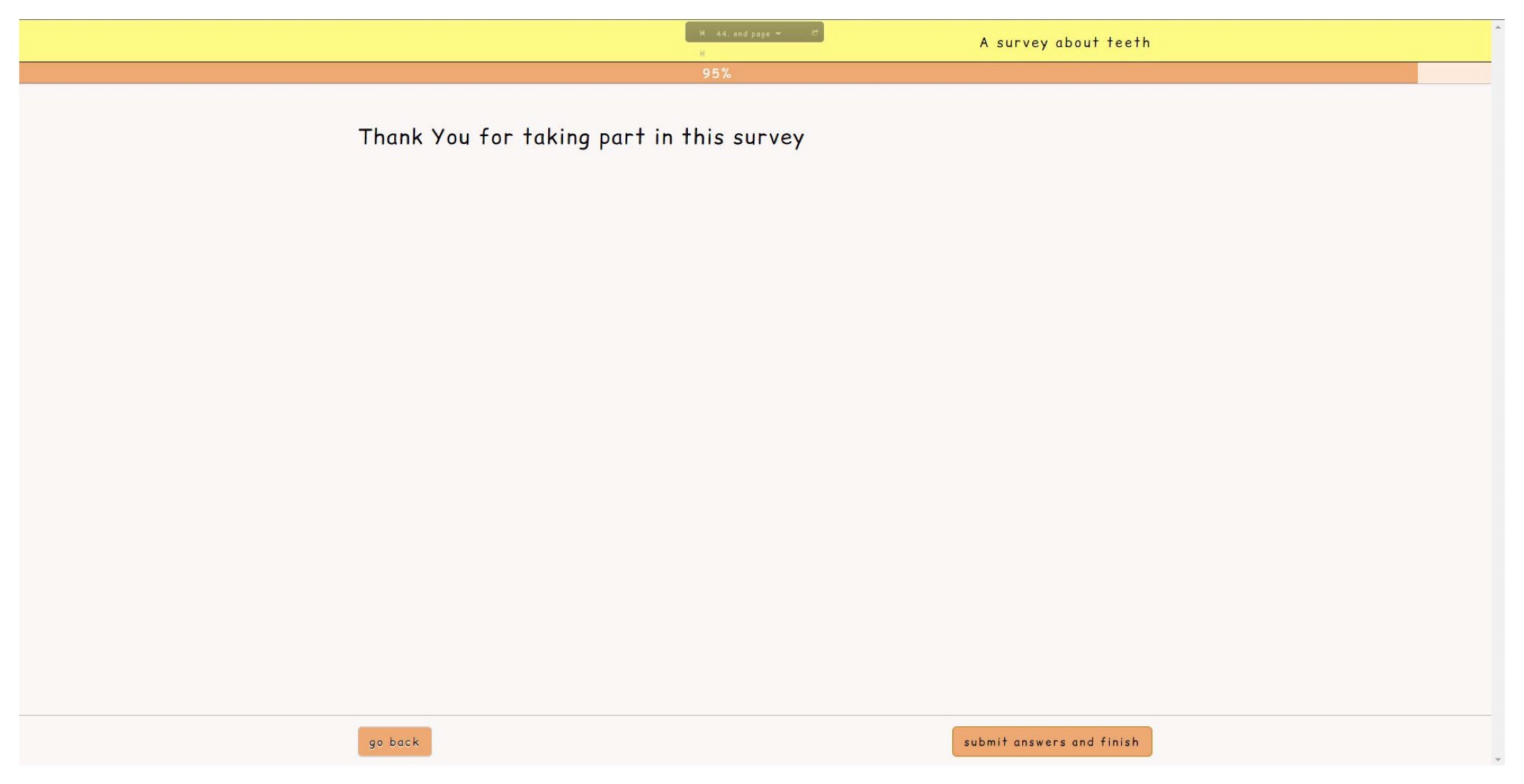

Supplement: Supplementary file 1 — Additional file 1. [file 41687_2021_302_MOESM1_ESM.docx]

####
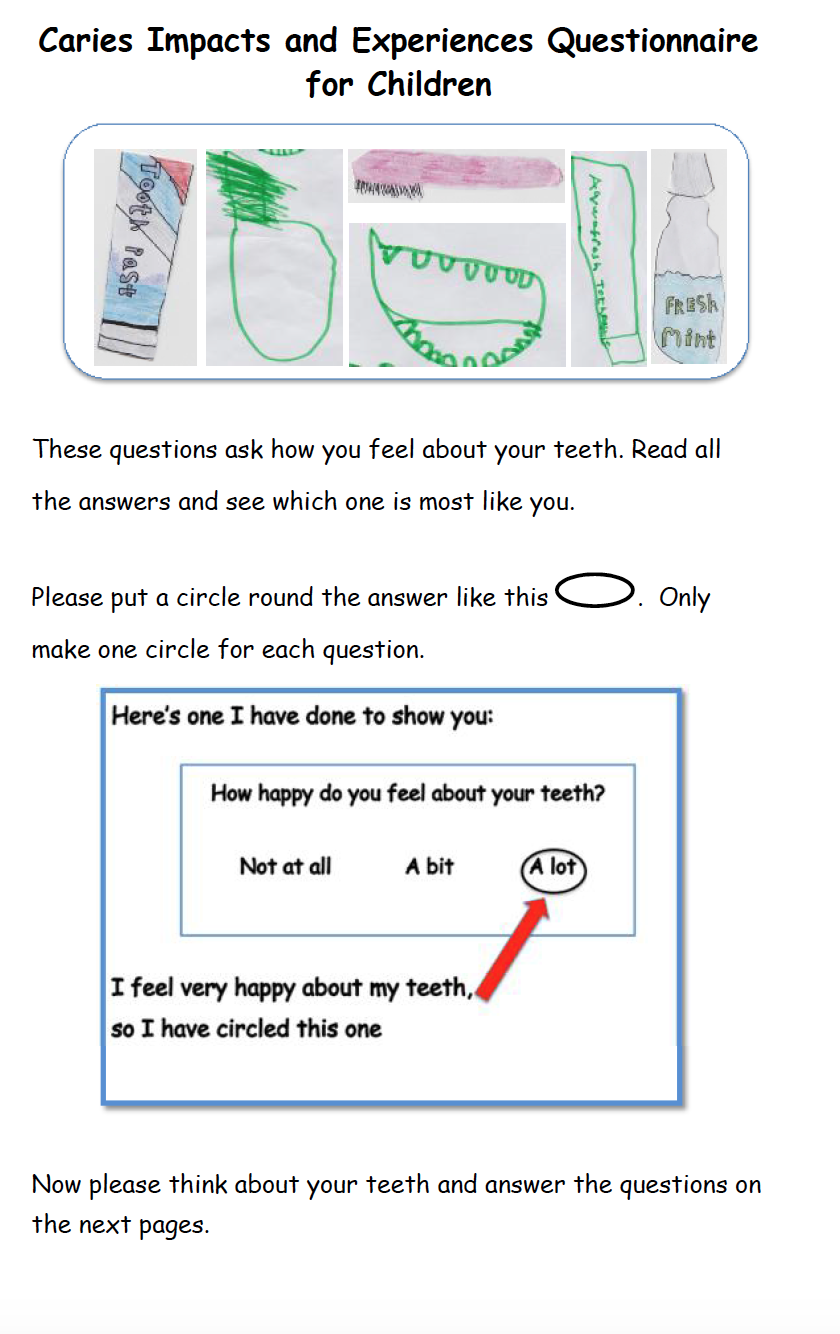


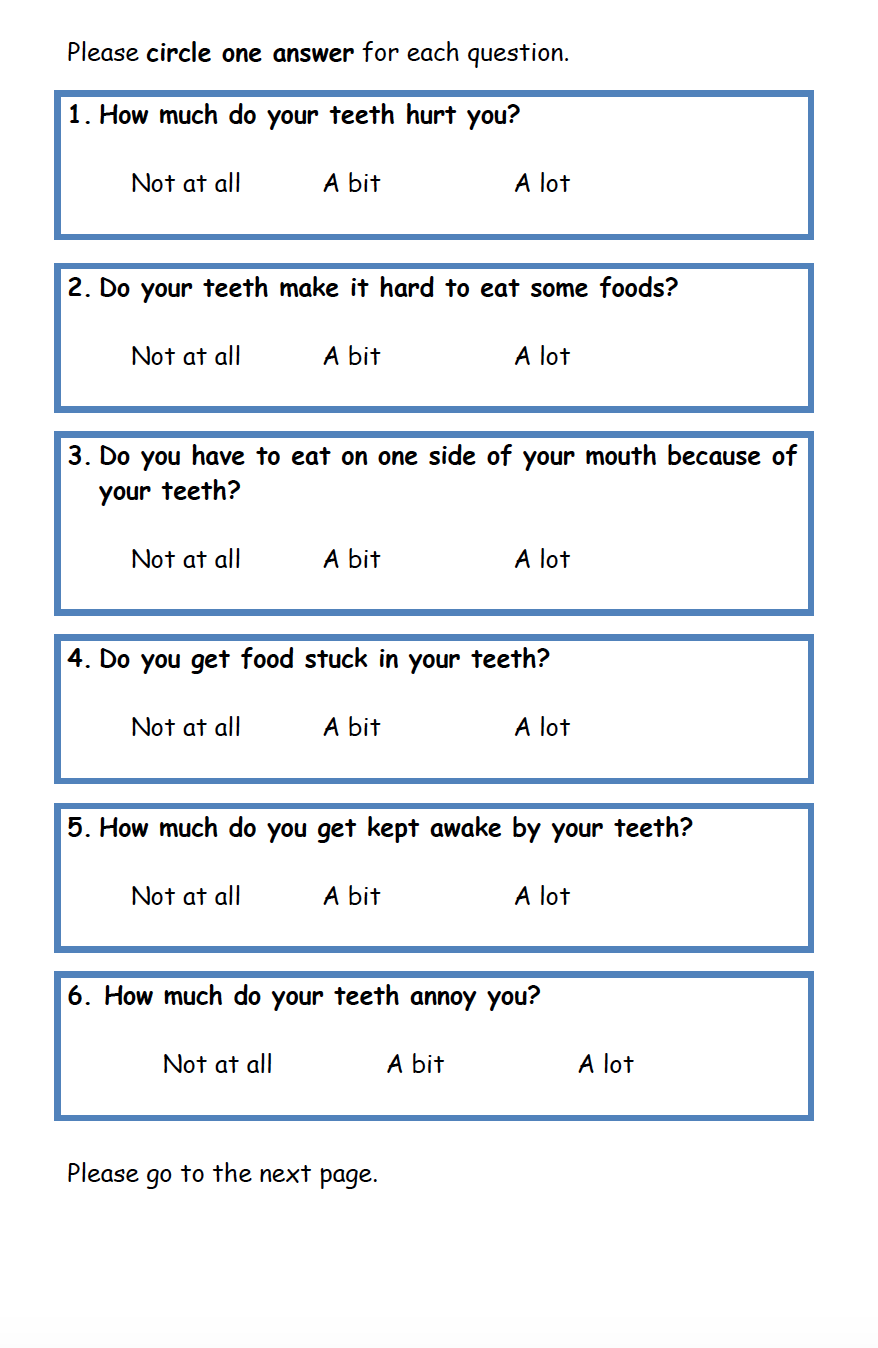


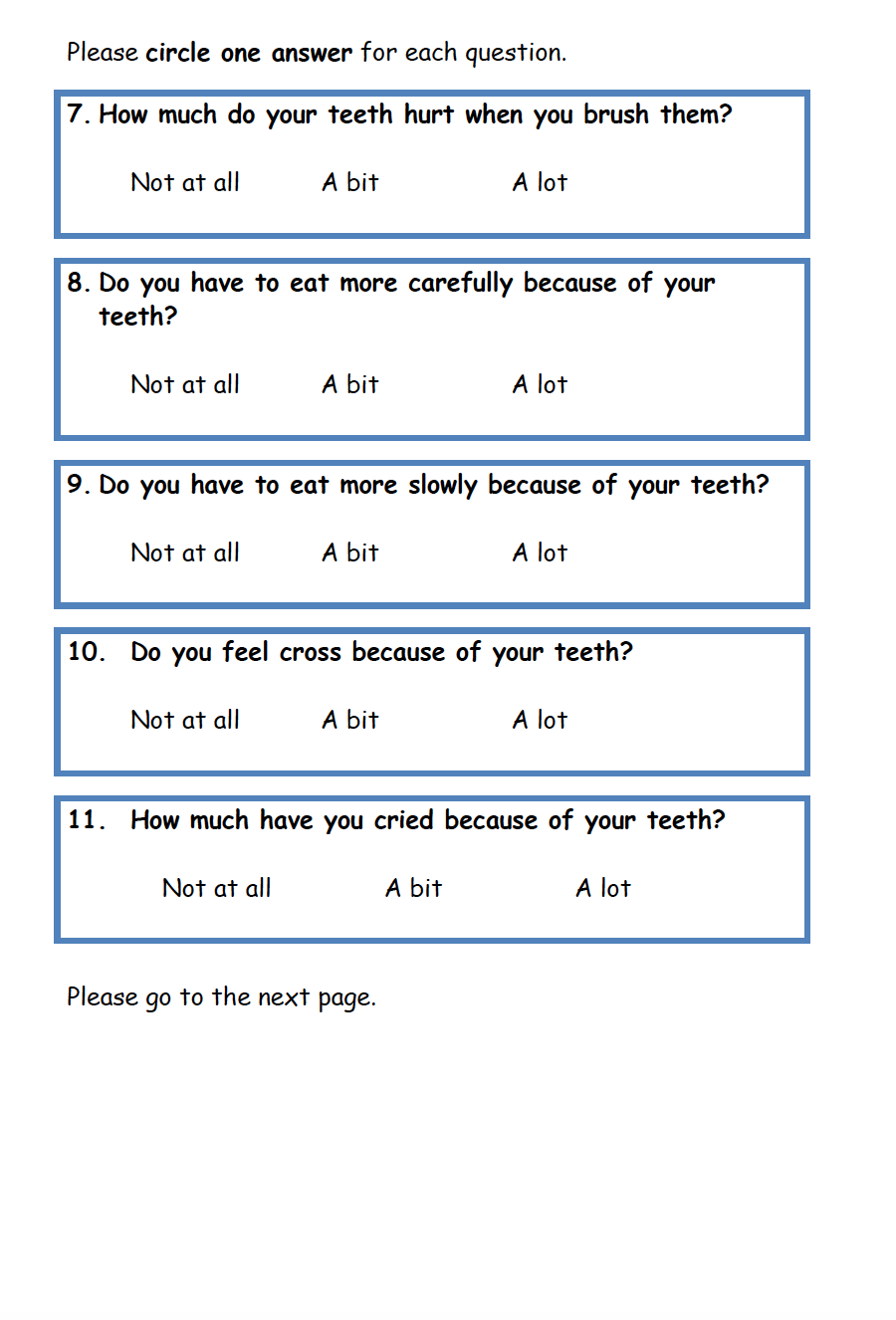


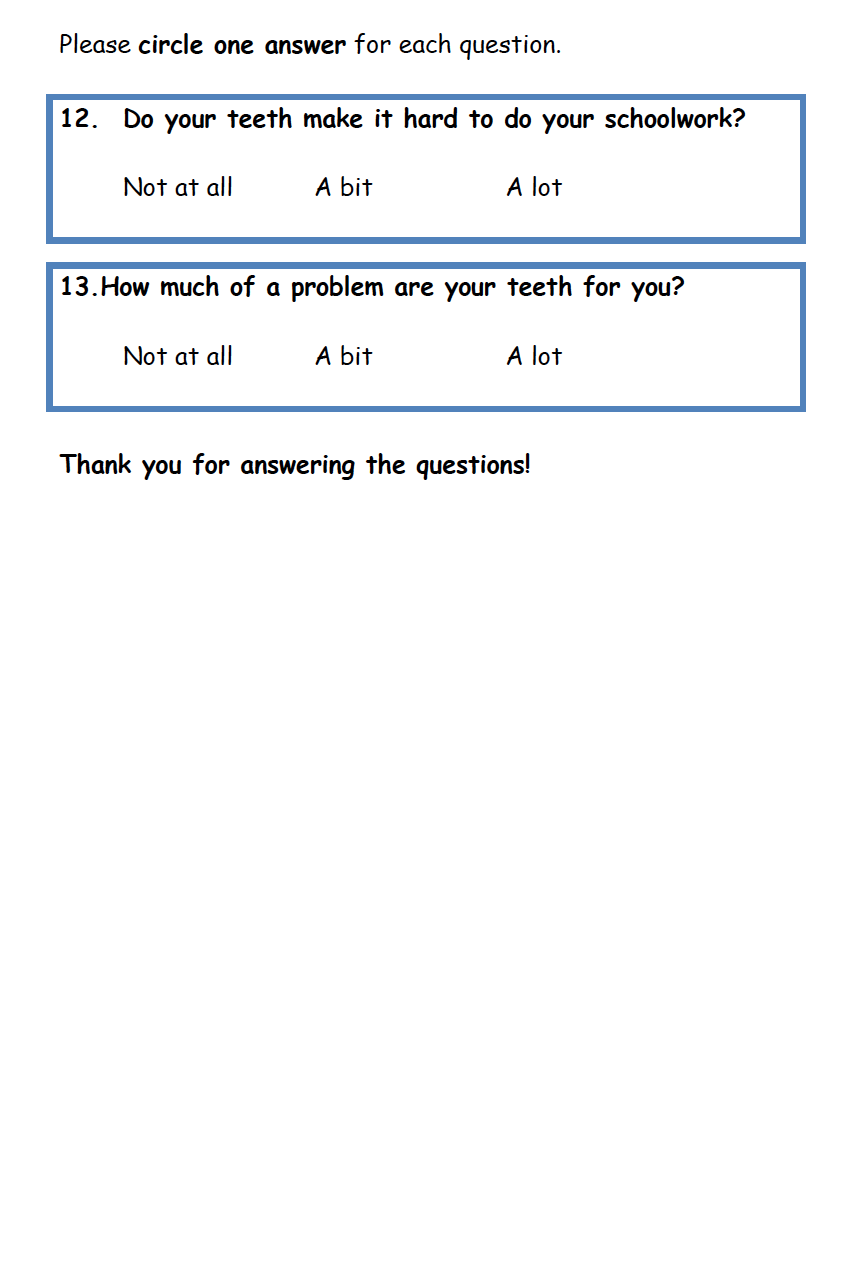

Supplement: Supplementary file 3 — Additional file 3. [file 41687_2021_302_MOESM3_ESM.docx]

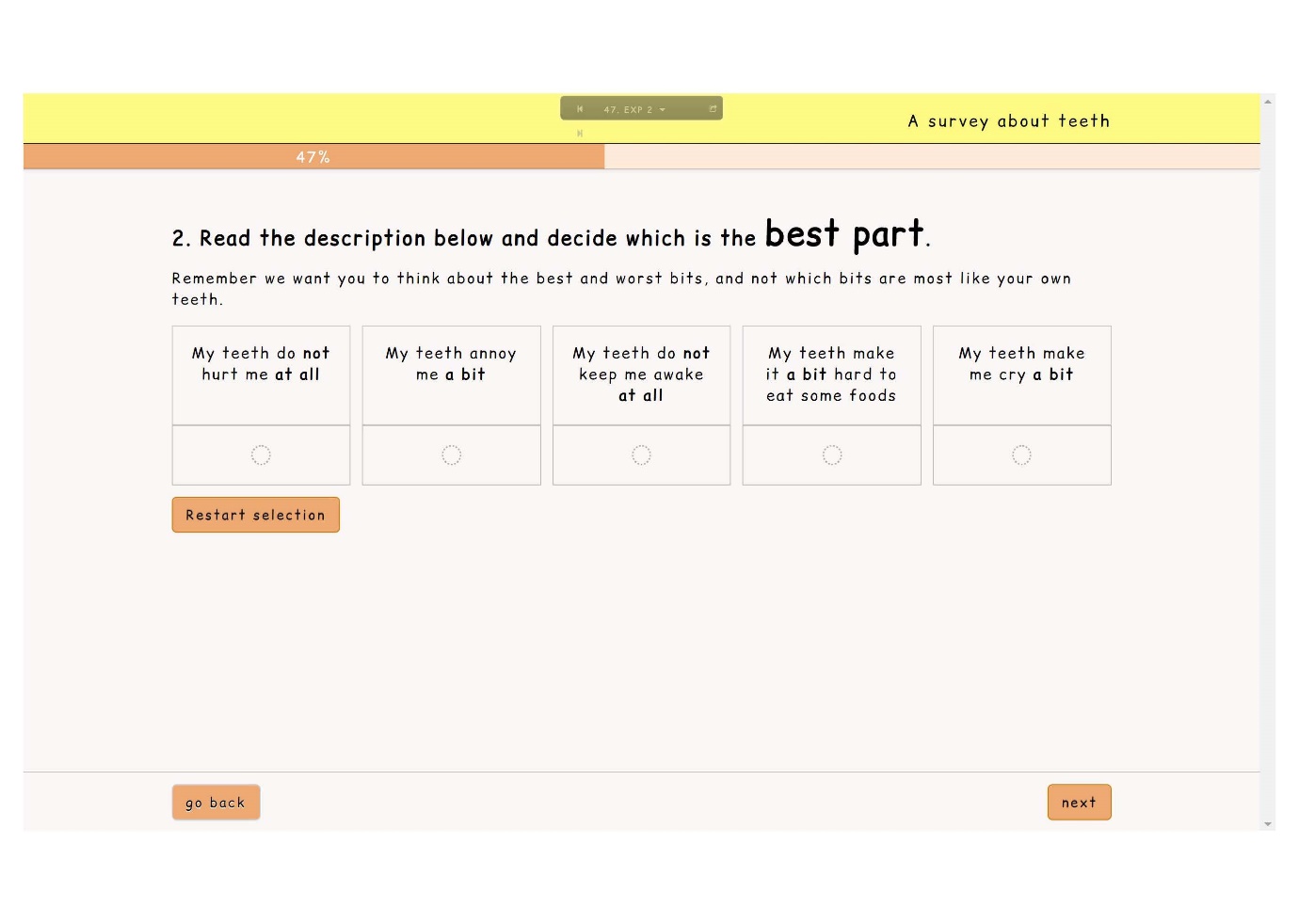

Supplement: Supplementary file 4 — Additional file 4. [file 41687_2021_302_MOESM4_ESM.docx]
