# Supplementary material for: Discrete choice experiments or best-worst scaling? A qualitative study to determine the suitability of preference elicitation tasks in research with children and young people
Source: J Patient Rep Outcomes. 2021 Mar 10;5:26. doi: 10.1186/s41687-021-00302-4 (PMC7947050; doi:10.1186/s41687-021-00302-4)
Supplement: Supplementary file 2 — Additional file 2. [file 41687_2021_302_MOESM2_ESM.docx]

*Supplement 1: Topic guide used to steer qualitative interviews with participants*

**Developing a caries-specific child-centred utility measure**

**(A survey about teeth: what do you think?)**

**Topic Guide**

**Introduction**

- Ensure participant has seen PIS
- Complete assent forms for participant
- Purpose is to find out what young people think about a survey about teeth
- Young people will be asked to fill in the survey. They will then be asked to ‘think aloud’ whilst completing it. They will also be asked some more questions about the survey at the end
- Interview will last as long as they wish, but on average 20 minutes
- Use of a digital recorder by researcher and then this will be written up
- Not a test, and no right or wrong answers (young person is the expert)
- Can ask for help with reading or filling in the survey at any time
- Doesn’t have to talk about anything they don’t want to
- Participation is voluntary (can change their mind and stop the interview at any point)
- Answers will be private

**Think Aloud task prompts**

- What are you thinking now?
- What are your thoughts about this question?
- Can you tell me more about that?

**Survey usability**

- What did you think about the survey?
- How did you find the survey?
- Were there any parts of the survey that were hard for you to understand?
- Were there any parts of the survey that were hard for you to answer?
- Can you see any problems with any of the questions?

**Type and number of task**

- Were there any questions in the survey that you didn’t like? Why didn’t you like them?
- Were there any questions in the survey that you did like? Why did you like them?
- Which style of question do you prefer and why? (Point to DCE and BWS tasks)
- How many of these type of questions do you think you could manage before you lose concentration or get bored?

**Practice questions and information about caries**

- During the survey you were asked to complete two practice questions. What did you think about these questions? Did you think they were helpful or not?
- Do you think more practice questions would help you or not? Do you think a different practice question would be better? Would a practice question that is not about teeth be useful or not?
- In the survey, you were given some information about tooth decay (holes). What did you think about this information? Did you find it useful? Is there anything you would want to change or add to this information?

**Next steps**

- Thank the participant
- Reassurance again about confidentiality/privacy
- Explain that findings will be used to develop a survey that will help us find out which dental treatments are value-for-money
- Inform the child that they will receive a copy of the final report through their school
- Give the participant a gift voucher and ask them to sign reciept
